# Machine Learning Classification Model for Functional Binding Modes of TEM-1 β-Lactamase

**DOI:** 10.3389/fmolb.2019.00047

**Published:** 2019-07-09

**Authors:** Feng Wang, Li Shen, Hongyu Zhou, Shouyi Wang, Xinlei Wang, Peng Tao

**Affiliations:** ^1^Department of Chemistry, Center for Scientific Computation, Center for Drug Discovery, Design, and Delivery (CD4), Southern Methodist University, Dallas, TX, United States; ^2^Department of Industrial, Manufacturing, and Systems Engineering, University of Texas at Arlington, Arlington, TX, United States; ^3^Department of Statistical Science, Southern Methodist University, Dallas, TX, United States

**Keywords:** TEM-1 **β**-lactamase, functional binding modes, structural analysis, random forest classification, machine learning, molecular dynamics

## Abstract

TEM family of enzymes is one of the most commonly encountered β-lactamases groups with different catalytic capabilities against various antibiotics. Despite the studies investigating the catalytic mechanism of TEM β-lactamases, the binding modes of these enzymes against ligands in different functional catalytic states have been largely overlooked. But the binding modes may play a critical role in the function and even the evolution of these proteins. In this work, a newly developed machine learning analysis approach to the recognition of protein dynamics states was applied to compare the binding modes of TEM-1 β-lactamase with regard to penicillin in different catalytic states. While conventional analysis methods, including principal components analysis (PCA), could not differentiate TEM-1 in different binding modes, the application of a machine learning method led to excellent classification models differentiating these states. It was also revealed that both reactant/product states and apo/product states are more differentiable than the apo/reactant states. The feature importance generated by the training procedure of the machine learning model was utilized to evaluate the contribution from residues at active sites and in different secondary structures. Key active site residues, Ser70 and Ser130, play a critical role in differentiating reactant/product states, while other active site residues are more important for differentiating apo/product states. Overall, this study provides new insights into the different dynamical function states of TEM-1 and may open a new venue for β-lactamases functional and evolutional studies in general.

## Introduction

Antibiotic resistance against almost all the existing antibiotics presents a major risk to global health. Among many other factors, β-lactamases as a group of proteins that hydrolyze antibiotics play a key role in antibiotic resistance. The serine β-lactamases, which utilize a serine residue to hydrolyze the β-lactam ring-based antibiotics, and zinc based β-lactamases, are the two main groups of β-lactamases in general. Class A β-lactamases are one dominant subgroup in serine β-lactamases and are highly diversified. TEM-1, the most commonly encountered β-lactamase in Gram-negative bacteria, belongs to the Class A β-lactamases (Bradford, 2001). The structure and potential catalytic mechanisms of TEM-1 have been studied extensively as a model system of Class A β-lactamases (Lamotte-Brasseur et al., 1991, 1999; Jelsch et al., 1992; Fonzé et al., 1995; Maveyraud et al., 1998; Petrosino et al., 1998; Minasov et al., 2002; Díaz et al., 2003; Hermann et al., 2003; Golemi-Kotra et al., 2004; Roccatano et al., 2005; Savard and Gagné, 2006; Doucet et al., 2007). The catalytic mechanism of TEM-1 can be divided into acylation and deacylation steps using penicillin as an example. The acylation step leads to an acylenzyme Michaelis-complex intermediate with a covalent bond formed between the Ser70 residue and ring opening product of penicillin β-lactam ring. This covalent bond in the acylenzyme intermediate is further hydrolyzed during the deacylation step, leading to an ineffective β-lactam ring-opening product detached from the enzyme. Catalytic functions of key residues at and surrounding an active site have been investigated extensively with some ongoing controversy (Oefner et al., 1990; Herzberg and Moult, 1991; Lamotte-Brasseur et al., 1991, 1992, 1994; Strynadka et al., 1992, 1996; Matagne et al., 1998). The active site of TEM-1 contains several conserved residues that are important for catalysis: Ser70, Lys73, Lys234, Glu166, and Ser130 (Fisette et al., 2010). Here and in the rest of the article, the sequence numbering of Ambler et al. (1991) is used to be consistent with the general literature about TEM-1 (Savard and Gagné, 2006; Doucet et al., 2007; Fisette et al., 2010). It is also believed that some residues, including Asn170, Ala237, Ser235, and Arg244, help to stabilize the acylenzyme intermediate. Although not fully determined, the contribution of these residues to TEM-1 catalytic mechanisms have been investigated extensively (Zafaralla et al., 1992; Stec et al., 2005; Marciano et al., 2009; Stojanoski et al., 2015; Palzkill, 2018). In addition, an allosteric site consisted of helixes 11 (residue 219–226) and 12 (residues 271–289) of TEM-1 were proposed (Horn and Shoichet, 2004). Two novel inhibitors were reported to destabilize the TEM-1 at high temperature. The two inhibitors can bind to the allosteric site in TEM-1, which locates in between helices 11 and 12. The allosteric site is 16 Å away from the active site. It was proposed that TEM-1 conformational changes were transmitted by a key catalytic residue, Arg244 (Horn and Shoichet, 2004). In another study, the allosteric site of TEM-1 was further detected through binding with a β-lactamase inhibitor protein (BLIP). It was suggested that the connections between active site and allosteric site may be modulated by the helix 10 region (residues 218–230) and Tpr229 in TEM-1 (Meneksedag et al., 2013). The allosteric site helixes 11 and 12 were also proposed as a cryptic pocket formation of TEM-1 (Oleinikovas et al., 2016). In addition, the residues P226-W229-P252 were identified as a PWP triad to stabilize the helix 10 region (Avci et al., 2016, 2018).

One important aspect of TEM-1 for its function is dynamics. Therefore, the molecular dynamics (MD) simulations were carried out to characterize dynamical properties of TEM-1 binding with benzyl penicillin molecule. A so-called Ω loop spans residues 163 through to 180 (including the key Glu166 residue for catalysis), and forms one edge of the active site (Dideberg et al., 1987; Herzberg and Moult, 1987; Moews et al., 1990; Jelsch et al., 1993; Vanwetswinkel et al., 2000). Some earlier MD simulations showed that the Ω loop was rather stable even with the absence of the ligand (Díaz et al., 2003). The whole TEM-1 has also been shown to be unusually rigid with limited motions on the picosecond-to-nanosecond time scale through a nuclear magnetic resonance (NMR) spectroscopy study (Savard and Gagné, 2006). Through more extended simulations and NMR studies, a variety of motions displayed by Ω loop are revealed to be potentially important for catalysis (Fisette et al., 2010). Another simulation study of TEM-1 binding with benzylpenicillin suggested that a substrate binding led to increased flexibility of Ω loop while making TEM-1 globally more rigid (Fisette et al., 2012). In addition to benzylpenicillin as a substrate, simulations were also carried out for TEM-1 bound with another two antibiotics, amoxicillin and ampicillin, to reveal that even the subtle differences in chemical structures of ligands could also regulate the substrate recognition (Pimenta et al., 2013).

One overlooked aspect of TEM-1's function is the binding with antibiotics and their hydrolysis product. Penicillin, for example, could bind with TEM-1 as favorable substrate, while the hydrolysis product of penicillin needs to leave the binding pocket for the turnover of this enzyme. Given the rigidity and sensitivity of the TEM-1 structure to the ligand, the response of protein dynamics to the ligand, in different chemical states through catalysis, could be significant and important for its function, however, this remains under-appreciated. One of the reasons for this is probably due to the fast turnover rate, which does not allow for a reliable experimental probe of the protein binding with ligands during its quick catalytic cycles. MD simulations provide an alternative way to scrutinize the difference between the binding modes of protein with similar ligands. However, due to the rigidity of TEM-1 and the similarity between two ligands of interest, some special analysis tools would be necessary for the purpose of comparison.

Machine learning methods are computational tools that construct data-driven prediction models based on training data. In recent years, machine learning methods have been successfully applied in computational chemistry (Husic and Pande, 2018), including pharmaceutical data analysis (Burbidge et al., 2001), protein–ligand binding affinity prediction (Ballester and Mitchell, 2010; Decherchi et al., 2015) and MD simulations based on machine learning analysis of quantum-mechanical forces (Li et al., 2015; Cortina and Kasson, 2018; Shcherbinin and Veselovsky, 2019). Recently, we have introduced two widely applied machine learning algorithms, a decision tree and an artificial neural network, to build classification models to differentiate two allosteric states of the second PDZ domain (PDZ2) in the human PTP1E protein as a dynamics-driven allosteric protein (Zhou et al., 2018). Despite the lack of a significant conformational change between two states of PDZ2, it was demonstrated that both algorithms could build effective prediction models and provide reliable quantitative evaluation of the contributions from individual residues to overall difference between the two states.

In this study, we applied another machine learning algorithm, random forest, to build models. Random Forest (Breiman, 2001) is a supervised learning algorithm that relies on an ensemble method to create an entire forest of random uncorrelated decision trees, in order to achieve a more accurate and stable prediction. It has been found to be very useful in a wide scope of applications, due to its superior performance in classification and regression problems, as well as its ease of use and flexibility. The recognition of TEM-1 against ligands in different states is interrogated through simulations studies. The random forest method as an effective machine learning technique has been applied to analyze the simulations of TEM-1 in different binding states and evaluate the contribution from every residue and related secondary structures to the recognition of ligands in different states of TEM-1. Potential key residues could be identified based on their feature importance generated from the machine learning model of the simulation data of TEM-1 in different states. The TEM-1 hydrolysis mechanism is of great interest and has been subjected to extensive computational studies focusing on the TEM-1 active site or nearby residues (Díaz et al., 2001; Meroueh et al., 2005; Roccatano et al., 2005; Sgrignani et al., 2014). However, the potential contribution from protein dynamics in different states to catalysis has been largely overlooked. We hypothesize that TEM-1 in different catalytic states, including binding states with reactant and product, are differentiable and could provide further mechanistic details if subjected to appropriate analyses.

Therefore, the current study focuses on the development of classification models to differentiate dynamics of TEM-1 in different functional states and on obtaining information to correlate protein dynamics with individual residues regardless their positions relate to the active site. The dynamics of different states are compared with each other in the training process, governed by the random forest method. In the random forest method, the contribution from each residue to the overall classification model was measured as importance of features (Zhou et al., 2019). A higher importance value of a feature represented a higher contribution in classifying different functional states. Using the feature importance, important structures and residues identified by this computational study are also in agreement with previous studies of this enzyme. The analysis about active and allosteric sites of TEM-1 also sheds new light on the allosteric component of TEM-1 functions. The remainder of the paper is organized in four parts: computational methods, results, discussion, and conclusion.

## Computational Methods

### Molecular Dynamics (MD) Simulations

**Figure 1:**
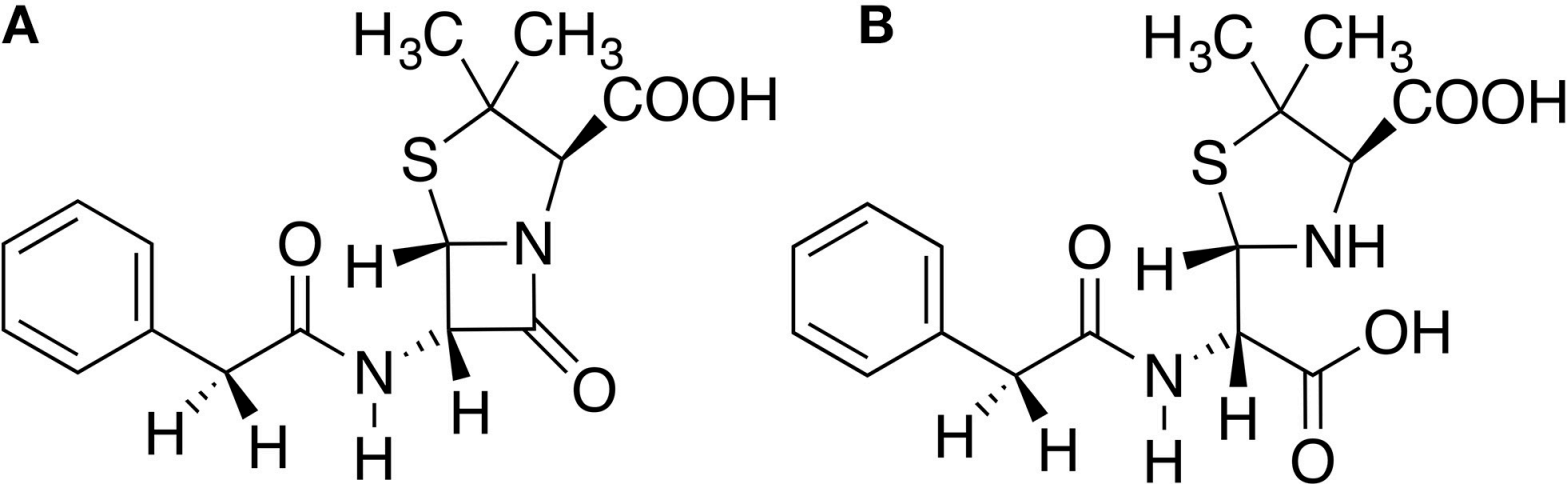
Chemical structures of **(A)** Benzyl penicillin, **(B)** the hydrolysis product of benzyl penicillin.

Three states of TEM-1 were subject to molecular dynamics (MD) simulations. TEM-1 bound with benzyl penicillin (Figure 1A) is referred to as the reactant state; TEM-1 bound with product of hydrolyzing benzyl penicillin (Figure 1B) is referred to as the product state, and TEM-1 alone without a ligand is referred to as the apo state. No crystal structure is available for TEM-1 binding with penicillin either as a reactant or product. The complex structure related to TEM-1 catalysis against penicillin with the best quality is an intermediate structure (PDB ID: 1fqg), which has been used for various computational studies. Therefore, this crystal structure was used to generate all three states of TEM-1, based on a hypothesis that equilibrium simulations could lead to sufficient sampling in these functional states. CHARMM molecular simulation program suite, version 40b1, was used to prepare and set up the systems (Halgren, 1992). Hydrogen atoms were added to the crystal structure of TEM-1 bound with benzyl penicillin using the hydrogen position construction facility (HBUILD) of the CHARMM. The benzyl penicillin ligand was removed to create the apo state of TEM-1. The benzyl penicillin structure was also modified using CHARMM internal coordinate editing functions to produce the benzyl penicillin hydrolysis product. CHARMM36 force field was used for TEM-1(Best et al., 2012). The CHARMM General Force Field (CGenFF) was generated for the benzyl penicillin and the benzyl penicillin hydrolysis product using online server ParamChem (https://cgenff.paramchem.org/). All systems are solvated in a water box using a TIP3P model with the addition of sodium and chloride ions to balance the charge and reproduce typical physiological ion concentrations.

The simulation boxes were subjected to 5,000 steps of the steepest descent energy minimization and further energy minimization using the adopted basis Newton-Raphson (ABNR) method until the total gradient of the system was lower than 0.02 kcal/mol•Å. Subsequently, the minimized simulation systems were subjected to 24 picoseconds (ps) isothermal-isobaric (NPT) ensemble equilibrium, gradually raising the temperature from 100 to 300 K. The system was then equilibrated via NVT ensemble MD simulations at 300 K. The time step for MD simulations is 2 fs, with all the bonds associated with hydrogen being fixed during the simulation using SHAKE method (Ryckaert et al., 1977). Periodic boundary condition was used in all simulations, and electrostatic interactions were calculated using the particle mesh Ewald method (Darden et al., 1993). For each state, five independent 100 ns NVT ensemble MD simulations were carried out as the production runs after 10 ns of equilibration. OpenMM simulation package was used to carry out the production MD simulations (Friedrichs et al., 2009; Eastman and Pande, 2015; Eastman et al., 2017).

### Analysis of MD Simulations

#### Root-Mean-Square Deviation (RMSD)

RMSD is used to measure the difference in conformation for each snapshot of the MD simulations from a reference structure. For a molecular structure represented by Cartesian coordinate vector *r*_*i*_ (*i* = 1 *to N*) of *N* atoms, the RMSD is calculated as the following:

(1) $$\begin{array}{r} \text{RMSD}=\sqrt{\frac{\sum_{i=1}^{N}{\left(r_{i}^{0}-Ur_{i}\right)}^{2}}{N}}, \end{array}$$

Where $r_{i}^{0}$ is the Cartesian coordinate vector of the *i*^th^ atom in the reference structure. The transformation matrix U is defined as the best-fit alignment between the TEM-1 structure along trajectories with respect to the reference structure.

#### Root-Mean-Square Fluctuation (RMSF)

RMSF is used to measure the fluctuation of conformation for each frame of the trajectories from the averaged structure.

(2) $$RMSF_{i}={\left[\frac{1}{T}\sum_{t=1}^{T}{\left|r_{i}\left(t\right)-r_{i}\right|}^{2}\right]}^{\frac{1}{2}},$$

Where T is the time period and $\bar{r_{i}}$ is the averaged position of atom *i* over the whole time period.

#### Principal Component Analysis (PCA)

For each state, PCA was performed by projecting each of the extracted 25,000 frames from five independent trajectories on the principal normal modes. The analysis was carried out using mdtraj package (McGibbon et al., 2015) and scikit-learn library in python (Pedregosa et al., 2011). PCA is a method to reduce the dimensionality of the motion of molecules. It can extract the dominant modes of the motion from a trajectory of molecular dynamic simulation. The normal modes for PCA (Jolliffe, 2011) were obtained through diagonalizing the correlation matrix of the atomic position in one trajectory. The correlation matrix element is calculated by

(3) $$\begin{array}{r} C_{ij}=\;\frac{c_{ij}}{\sqrt{c_{ii}c_{jj}}}=\;\frac{\langle r_{i}r_{j}\rangle -\langle r_{i}\rangle \langle r_{j}\rangle }{\sqrt{\left[\left(\langle r_{i}^{2}\rangle -{\langle r_{i}\rangle }^{2}\right)\left(\langle r_{j}^{2}\rangle -{\langle r_{j}\rangle }^{2}\right)\right]}}\;, \end{array}$$

Where *C*_*ij*_ is the Pearson correlation coefficient between atoms *i* and *j*.

The distributions of three TEM-1 states simulations in the PCA projection space are normalized and plotted as a density contour graph. The distribution density function was estimated by the Gaussian kernels (Scipy 1.2.1) (Turlach, 1993; Bashtannyk and Hyndman, 2001; Scott, 2015; Silverman, 2018).

#### Random Forest Model

The random forest classification was used in this study to develop classification models for the three states of TEM-1. The python package scikit-learn v0.20.3 was used to carry out the training and testing using this model. For each independent 100 ns simulation of all states, 5,000 frames were evenly extracted as the training and testing data. For each state, four simulations among five production runs were randomly selected as the training set with the remaining simulation used as the testing set. For each selected frame from the simulation, all the pairwise distances among the α carbons (Cα) of TEM-1 backbone are extracted as the features for training purpose. A total of 263 TEM-1 amino acid residues result in 34,453 pairwise distances as the training features. As a pre-step before the classification, the feature selection is carried out using the random forest classification model. Following a previous study to build feature selection using machine learning methods (Zhou et al., 2018), all features are pre-screened to select features accounting for 98.0% as total importance. The apo/product model has 901 features out of the total of 34,453 features. Similarly, after the feature selection, the reactant/product model has 1,170 features, the non-product/product model has 964 features and the apo/reactant model has 1,923 features for their classification models. The final classification models were developed using these preselected features. The number of preselected features for four training models with all preselected features are provided in the Supplementary Material.

A random forest algorithm was built on the decision tree models. First, training data was randomly divided into numerous sets and decision tree models were built based on each set. Then all the decision tree models were combined to generate final random forest classification model (Breiman, 2001; Geurts et al., 2006; Louppe, 2014). The random forest algorithm implemented in scikit-learn v0.20.3 (ensemble.RandomForestClassifier) was employed in this study. The number of decision trees generated in the random forest model (referred to as n_estimator) was varied for the best performance with the highest training and validation accuracy (Supplementary Figure 1). For each model, the number of decision trees to obtain the highest accuracy of validation was selected for the final classification model.

The random forest method was employed for two purposes in this study, including feature prescreening and classification model developing. In feature prescreening, the feature importance generated from preliminary random forest training process is assigned to each feature. All features are sorted based on their feature importance. The features with the sum of their importance accounting for 98% are selected for the final classification model. These pre-screened features of each classification model present in this study are listed in the Supplementary Material. The final classification models were trained using the pre-screened features and with new set of feature importance generated from the training process. The new set of feature importance is used for further analyses presented in this study.

#### Scores

In this study, the scores including accuracy, precision, recall, and F1 score were used to evaluate the performance of each classification model. The python package v0.20.3 (Pedregosa et al., 2011; Buitinck et al., 2013) was employed to generate these four scores. The accuracy score is defined as

(4) $$\begin{array}{r} accuracy=\;\frac{1}{N}\sum_{i=0}^{N-1}1\left(\hat{y_{i}}=y_{i}\right), \end{array}$$

where *N* is the number of samples, $\hat{y_{i}}$ is the predicted label and *y*_*i*_ is the true label for the *i*_*th*_ sample.

In a binary classification task, such as the classification models in this study with two labels, the predictions of the model are evaluated as the following. Positive/negative labels are used to reflect the prediction made by the model. True/false are used to represent whether the predicted labels correspond to the observed labels (real labels). Accordingly, precision, recall and F1 scores are defined as the following.

(5) $$\begin{array}{r} precision=\;\frac{tp}{tp+fp}, \end{array}$$

(6) $$\begin{array}{r} recall=\;\frac{tp}{tp+fn}, \end{array}$$

(7) $$\begin{array}{r} F1=2\frac{precision^{*}recall}{precision+recall}, \end{array}$$

Term *tp* (true positive) represents the situation that the model gives positive prediction and the observed label is indeed positive. Term *fp* (false positive) represents that the model gives positive prediction, but the observed label is negative. Term *fn* (false negative) represents that the model gives negative prediction, but the observed label is actually positive. F1 score is a weighted mean of the precision and recall.

#### Feature Importance

The importance of each feature is generated by random forest algorithm based on Gini impurity (Equation 8). A higher importance represents a more important feature in distinguishing different states. The Gini importance implemented in python package scikit-learn v0.20.3 was used in this study and briefly introduced in the Equations (8–12) as the following.

The feature importance was calculated as Gini impurity:

(8) $$\begin{array}{r} Gini\;impurity=\;\sum_{i=1}^{C}-f_{i}\left(1-f_{i}\right), \end{array}$$

where *f*_*i*_ is the frequency of a label at a node, and C is the number of labels.

In the random forest models, many decision trees are constructed for training purpose. All the predictions from these individual trees are collected to make the final random forest classification model. The importance (*n*_*j*_) of a node *j* in each decision tree was represented by Gini impurity:

(9) $$\begin{array}{r} n_{j}=\;w_{j}C_{j}-\;\sum_{1}^{m}w_{m\left(j\right)}C_{m\left(j\right)}, \end{array}$$

where *w*_*j*_ is the weighted number of samples reaching node *j*, C_*j*_ is the impurity value of node *j*, and m is the number of child nodes of the tree.

The feature importance of feature *i* on decision tree is calculated as:

(10) $$f_{i}=\;\frac{\sum_{1}^{s}n_{j}}{\sum_{k\in all\;nodes}n_{k}},$$

where *s* is the times of node *j* split on feature *i*.

The normalized feature importance in a decision tree is calculated through:

(11) $$norm\;f_{i}=\;\frac{f_{i}}{\sum_{j\;\in all\;features\;in\;a\;tree}f_{j}},$$

The final feature importance in random forest classification is calculated as:

(12) $$F_{i}=\;\frac{\sum_{j\in all\;trees}norm\;f_{j}}{N},$$

where *norm f*_*i*_ is the normalized feature importance values of a decision tree, *N* is the total number of trees (Breiman, 2001; Geurts et al., 2006; Pedregosa et al., 2011; Louppe, 2014).

In our classification models, the features are pairwise Cα distances. To evaluate the importance of each amino acid residue, all the feature importance of the pairwise distances relating to each residue are summed up and divided by two to generate the importance of a residue. Then the total importance of 263 residues were accumulated and the importance percentage of each residue could be calculated based on the total importance. The value of importance percentage represents the ability of a residue to differentiate three states. In other words, the importance could help to evaluate the contribution from a residue to differentiate three states in dynamic motions.

## Results

### TEM-1 Three States Simulations Analysis

**Figure 2:**
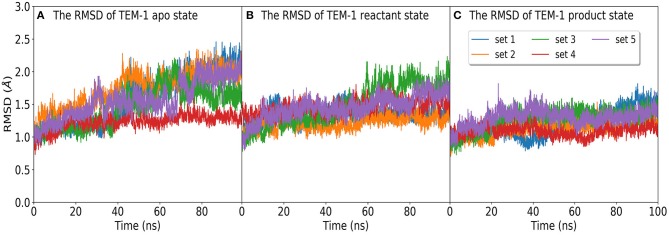
The RMSD distribution of molecular dynamics simulations of TEM-1 in **(A)** apo state, and binding with benzyl penicillin in **(B)** reactant and **(C)** product states. In each state, the RMSD are calculated in five independent 100 ns simulations labeled as set 1 to set 5.

The time evolution of the RMSD of TEM-1 in five independent simulation sets in apo, reactant, and product states are plotted in Figure 2. All RMSD values were calculated with reference to the TEM-1 crystal structure. The averaged RMSD values are 1.5, 1.3, and 1.1 Å for the apo, reactant, and product states, respectively. The plots suggest that the TEM-1 is rather stable with low RMSD fluctuations in all three states. Among three states, the apo state displays the highest TEM-1 fluctuation, and the product state displays the lowest TEM-1 fluctuation. To address the concern of the simulation convergence, we also calculated the accumulative entropy of TEM-1 in each state along each independent simulation (Supplementary Figure 2). All three states display clear convergence tendency in each simulation.

**Figure 3:**
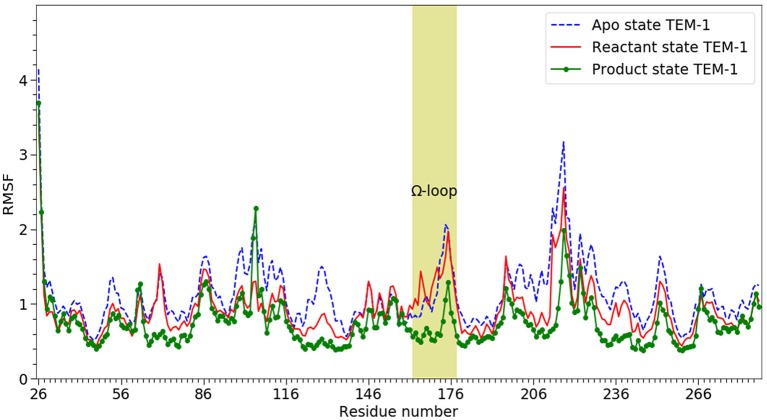
The RMSF of α-carbons (Cα) from 26 to 288 on TEM-1 β-lactamase in apo (blue dash line), reactant (red line) and product states (dot line), Ω loop (residue 163–180) highlighted. All three states have overall similar distribution but with significant difference. The product shows the lowest overall RMSF. The apo state show the highest overall RMSF.

RMSF of individual residues was calculated for each state using all five simulations and plotted in Figure 3. In agreement with the RMSD results, TEM-1 in the apo state has the highest fluctuation for most part of the protein (blue dashed line in Figure 3). However, TEM-1 in both the reactant and product states also displays higher fluctuation than the apo state in certain part, revealing that the binding with ligands and the type of ligand do exert a subtle impact on protein dynamics.

**Figure 4:**
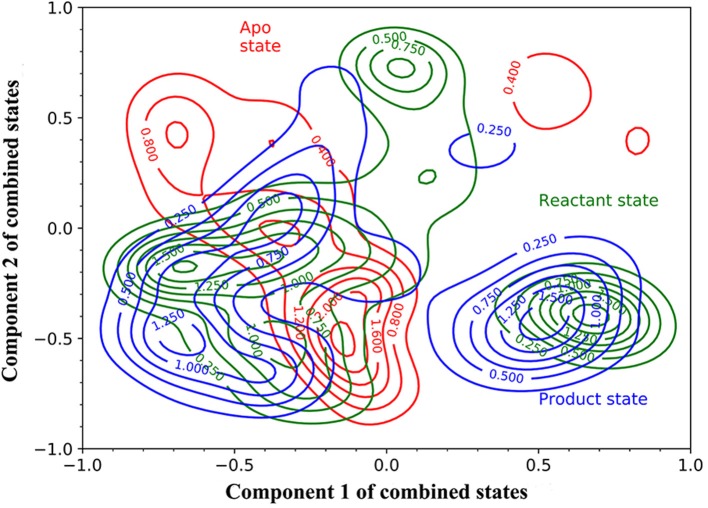
The projection of the simulations of TEM-1 in apo (red), reactant (green) and, product (blue) states onto Component 1 and Component 2 of combined states. Components 1 and 2 are the first and second components from the principal component analysis (PCA) based on the simulations of all three states. The projection on to components 1 and 2 are normalized.

Then, we carried out PCA using all 15 simulations from three states as an attempt to develop a model differentiating three states of TEM-1. The simulations of each state are projected onto the surface as contour plots with normalization using the first principal component (PC1) and second principal component (PC2) (Figure 4). Overall, all three states largely overlap with each other on the PC1/PC2 surface, and each state has two or three minima, which are referred to as attraction basins. The reactant and product states cover similar areas and largely overlap with each other, with their attraction basins close to each other. The apo state has different attraction basins and has much narrower distribution than the other two states. The PCA results reflect that the TEM-1 structure is generally rigid without significant global conformational change. However, the subtle differences among the distributions of TEM-1 in different states in the PCA space do indicate the shift in population of TEM-1 in different binding states. The following analysis using the random forest model provides more insight into these subtle differences.

### Random Forest Model

**Figure 5:**
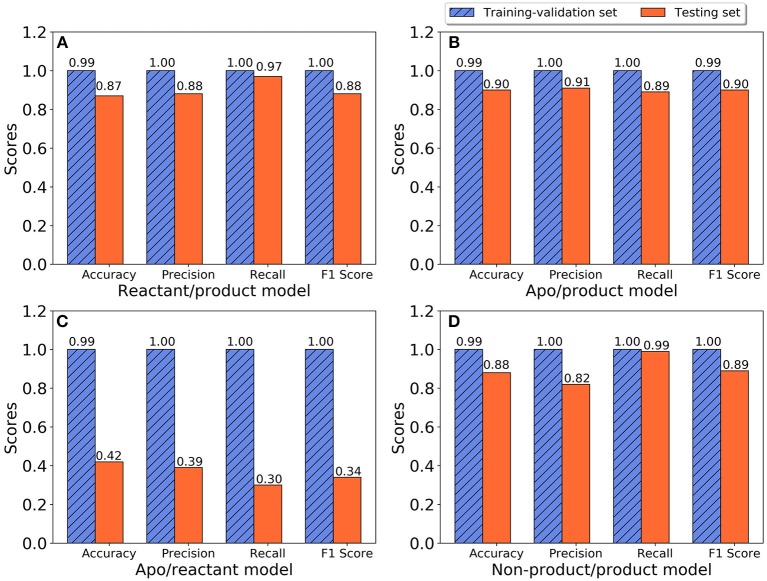
The performance of random forest classification models in accuracy, precision, recall, and F1 scores for training-validation set (blue shadow) and testing set (red). **(A)** Reactant and product states model; **(B)** Apo and product states model; **(C)** Apo and reactant states model; **(D)** Non-product and product states model.

The training and testing results of the random forest model for all three states, including accuracy, precision, recall, and F1 scores, are plotted in Figure 5. Classification models were developed to differentiate between apo and product states, reactant and product states, non-product (combining the apo and reactant states) and product states, as well as between apo and reactant states. For the classification model to differentiate the reactant and product states, the training with cross-validation provides high performance, and testing provides better than 87% accuracy in all categories (Figure 5A), suggesting that the TEM-1 reactant and product states are highly differentiable using the Cα pairwise distances as protein structural information. Slightly better scores are obtained for the classification model to differentiate the apo and product states (Figure 5B). These results show that the TEM-1 in the product state is clearly distinguishable from TEM-1 in the apo and reactant states. However, distinguishability between the apo and reactant states of TEM-1 is significantly lower than the first two pairs (Figure 5C), suggesting that these two states share significant similarity in terms of protein backbone structural distributions represented as Cα pairwise distances. To further test this, both apo and reactant states are combined together to be considered as non-product state vs. product state. A classification model differentiating the non-product and the product states is built with cross-validation performance measures close to 100% and testing performance measures ranging between 82 and 99% (Figure 5D), similar to the models for apo/product and reactant/product pairs.

As part of preliminary study, two other widely applied machine learning methods, artificial neural network and support vector machine methods, were also applied to develop classification models for TEM-1. Both methods produced models with performance worse than random forest model (Supplementary Figures 3, 4). In addition, the random forest method provides importance numerical value for each feature, which could be used to search for key residues and functional groups in protein structure. Therefore, the remainder of the study focuses on random forest model result.

### Secondary Structures Contribution

In random forest classification models, each Cα pair is given an importance value reflecting its contribution for the classification model. These values could be used to evaluate, to some extent, the importance of individual amino acid residues. We first used these values to evaluate the contribution of secondary structures in TEM-1, with regard to the differences among different states. For each secondary structure, all the importance values associated with residues in that structure are summed together and divided by two as the overall importance. Three well performing classification models, apo/product, reactant/product, and non-product/product, are used for this comparison purpose. The TEM-1 structure is divided into β**-**sheets, α-helices, coils and turns as secondary structures and the residues inclusive in these structures. The β**-**sheet and α-helices of TEM-1 are defined in a previous study (Savard and Gagné, 2006), and are commonly used in general literatures of TEM-1 (Simm et al., 2007; Fisette et al., 2010, 2012). The definition of coils and turns in the database of secondary structure assignments (DSSP) are used in this study (Kabsch and Sander, 1983). There are some coils and turns with just one or two residues. Some of them have small importance values. For simplification, when such a short coil or turn is adjacent to another coil or turn, they are combined as a new coil or turn structure for analysis. However, if a short coil or turn is between β**-**sheets or α-helices, it was kept by itself.

**Figure 6:**
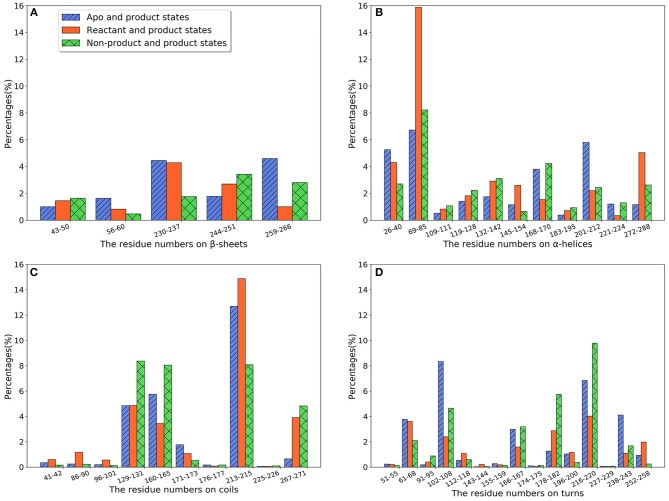
The total feature importance of individual secondary structure in TEM-1: **(A)** β-sheets; **(B)** α-helix; **(C)** Random coils; **(D)** Turns. Each secondary structure is labeled by residue number. There are five β-sheets and 11 α-helices with varying sizes in TEM-1 structure.

**Figure 7:**
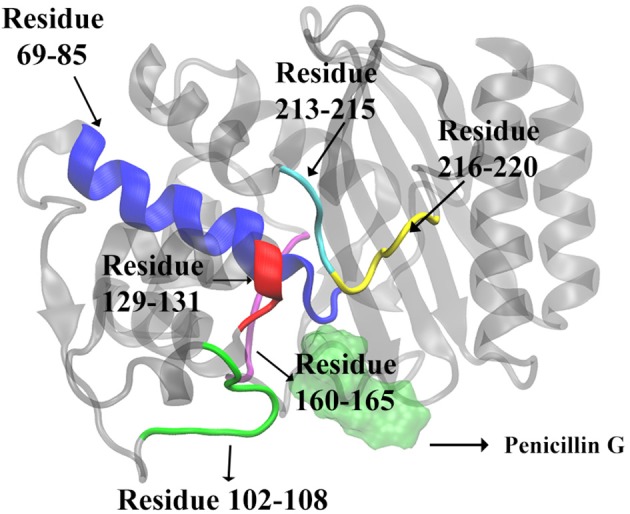
The secondary structures of TEM-1 with significant total feature importance. Residues 69 through 85 as α-helix (blue), residues 213 through 215 as random coil (cyan); residues 216 through 220 as turn (yellow); Residues 129 through 131 as random coil (red), residues 102 through 108 as turn (green), residues 160 through 165 as random coil (purple). The ligand penicillin-G molecule is also illustrated as green transparent surface. The residue 160–165 is behind of the residue 69–85 in this view.

We further calculate the importance of individual secondary structures and plot it in Figure 6. All five β-sheets in TEM-1 have importance values lower than 5% (Figure 6A), indicating that the β sheets may not play an important role, with regard to ligand binding. There are 11 helices with varying lengths in TEM-1. Most helices have low importance (Figure 6B). The only exception is helix (69–85), which has overall importance close to 16% in the reactant/product model (Figure 6B), and also one of the helices around the active site of TEM-1 (Figure 7 green transparent surface).

There are 10 short fragments being considered as random coils in TEM-1. Among this, residues 213–215 coil shows the highest importance in all three models (Figure 6C), which is illustrated and highlighted as cyan structure in Figure 7. The second important coil is residues 129–131, with three residues accounting for more than 8% importance in the non-product/product model and around 5% in the other two models. Both 213–215 and 129–131 (highlighted as red structure in Figure 7) coils are adjacent to the active site.

There is a total of 15 turn structures in TEM-1, some with significant difference among three classification models. The importance of the residue 216–220 turn (highlighted as yellow structure in Figure 7) is the highest on average among all turn structures, followed by residues 102–108 turn (highlighted as green structure in Figure 7). Both turns are positioned as gate to cap the TEM-1 active site.

**Figure 8:**
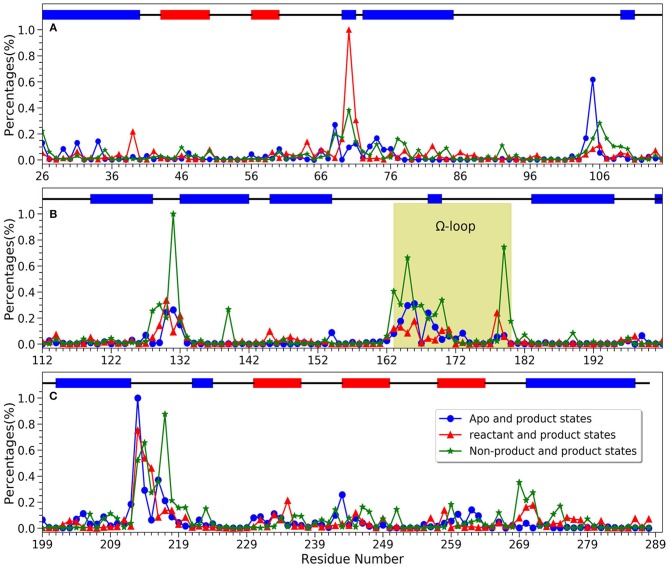
The accumulative feature importance of each residue in TEM-1. The blue circles represent the apo and product states classification model, the red triangles represent the reactant and product states classification model, and the green stars represent the non-product and product states classification model. On the top of each sub-figure, the β-sheets and α-helices are labeled as red and blue rectangles, respectively. Ω-loop is highlighted in yellow. **(A)** Residues 26 through 115; **(B)** Residues 112 through 202; **(C)** Residues 199 through 288.

For a better understanding of each residue, the mapping of importance percentage of each residue in TEM-1 obtained from the machine learning training process is plotted in Figure 8 (divided into three parts A, B, and C). The serial numbers of residues from the PDB file that start from 26 to 111 are used in Figure 8A, from 112 to 198 are used in Figure 8B and from 199 to the end 288 are used in Figure 8C. The overall distributions of TEM-1 individual residue importance based on different classification models resemble each other. Residue 213 has the highest percentage (9.3%) in the apo/product model (Figure 8C), which is also the highest percentage for a single residue among all three models. In reactant/product model, residue 70 has the highest percentage as 8.4% (Figure 8A). In all three models, residues 67–73, 103–107, 127–135, 162–171, 176–182, and 210–220 have relative high importance percentages in all three models. Interestingly, these residue regions were proposed to undergo conformational changes in a previous NMR study (Savard and Gagné, 2006).

**Figure 9:**
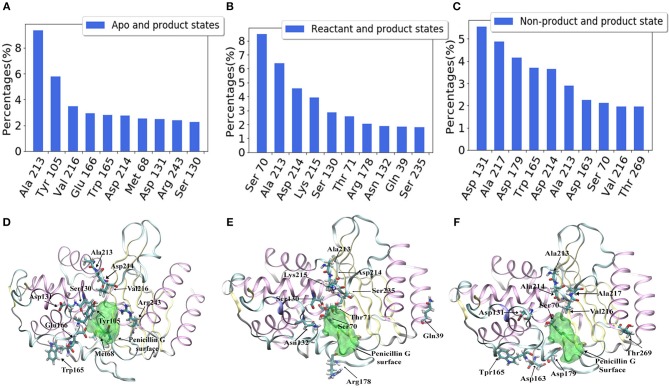
Top 10 residues with the highest feature importance in TEM-1 β-lactamase based on classification models: **(A)** Apo/product model, **(B)** Reactant/product model, **(C)** Non-product/product model; The structures of top 10 residues with the highest feature importance and their positions comparing with the active site of TEM-1 in each model: **(D)** apo/product model, **(E)** reactant/product model, and **(F)** non-product/product model. The ligand penicillin-G molecule is also illustrated as green transparent surface. The active site of TEM-1 is the pocket holding the penicillin-G molecule.

For each model, the top 10 residues with the highest percentages are listed and illustrated with the TEM-1 structure in Figures 9A–F. Most of the key residues identified through the classification model are not on either helices or strands secondary structures. However, few active site residues are among the top 10 residues (illustrated in green in Figures 9D–F). The percentages of active site residues are significantly different, which is plotted for all three models (Supplementary Figure 5). Ser70 from the TEM-1 active site has significantly high importance in the reactant/product model. Ser70 in the other two models, and all other active site residues, only display importance lower than 3%. These are in the agreement that the TEM-1 active site is generally rigid for the purpose of catalysis.

**Figure 10:**
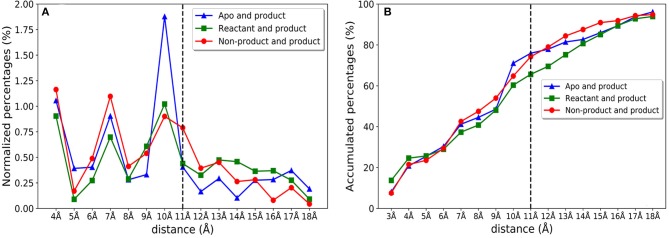
The feature importance of residues with reference to the distance from active site for apo/product, reactant/product, and non-product/product classification models. **(A)** The normalized feature importance of residues within certain distance from active site (using 1 Å window). For example, the importance percentage 0.25% in 5 Å represents the importance percentage of all residues located in distance range 4–5 Å away from the active site; **(B)** Accumulative feature importance of residues with a certain distance from the active site. For example, the importance percentage of 5 Å represents the importance percentage including all the residues within 5 Å away from the active site.

We further investigate the distribution of residues importance with reference to the active site. The importance of residues lying within a certain distance range (i.e., between 4 and 5 Å) from the active site residues are accumulated and normalized by the number of residues within a distance range, which is shown in Figure 10A. There are clearly three peaks of importance for the shells around 4, 7, and 10 Å away from the active site. The sums of importance of residues away from the active site region in the three models are plotted in Figure 10B. The accumulative importance of residues surrounding the active site is smoothly increasing along the distance.

### The Conformational Analysis

In three states classifications, the key residues are identified based on the feature importance obtained from the classification models. However, the conformational changes in three states are very important for detecting the catalytic mechanism of TEM-1 bound with penicillin G complex. Therefore, further conformational analysis is carried out based on the selected key residues with top feature importance. Among the top 10 residues based on their accumulative feature importance, Tyr105 as a gatekeeper of the active site could stabilize the ligand binding (Doucet and Pelletier, 2007; Doucet et al., 2007). However, the interaction between Asn132 and Tyr105 may perturb the stabilizations (Wang et al., 2002). And a mutant of Asn105 has been proposed to create disruptive steric clashes with Asn132 and destabilize the ligand binding (Doucet and Pelletier, 2007). Asn132 is also a special residue, which was proposed to provide additional space for active site (Swarén et al., 1998). Therefore, the distance between Cα atoms of Tyr105 and Asn132 was selected for further analysis to reveal detailed conformational change relevant to functional states. In addition, the interaction between Lys73 and Asn132 was reported as important residues for TEM-1's catalytic function (Swarén et al., 1998). Accordingly, the Cα atoms distance between Lys73 and Asn132 is subjected to further analysis in this study. Two residues Gln39 and Thr269 among the top 10 residues are distal from the active site. Thr269 is really close to the allosteric site Helices 12 (Residue 272–288) identified in previous study (Horn and Shoichet, 2004). To reveal potential correlation between the active site and Gln39 as well as Thr269, the Cα atoms distance from Ser70 as the center of active site to these two residues are also subjected to further analysis.

**Figure 11:**
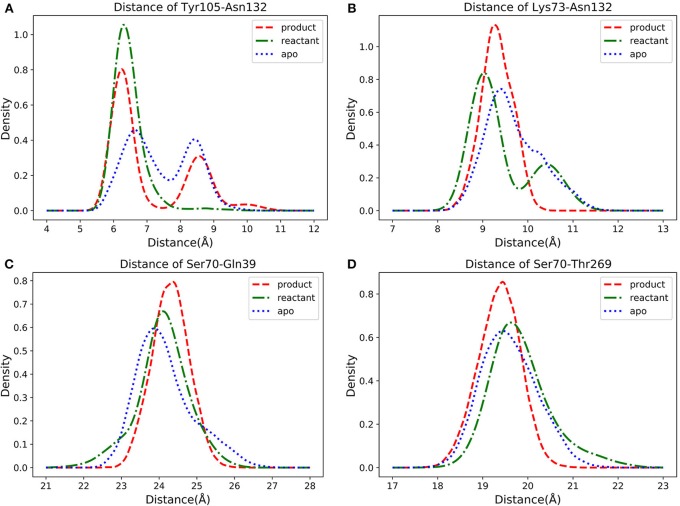
The density distributions of pairwise α carbon atoms distance in apo (blue dot line), reactant (green dot dashed line) and product (red dashed line) states: **(A)** Tyr105 and Asn132, **(B)** Lys73 and Asn132, **(C)** Ser70 and Gln39, **(D)** Ser70 and Thr269.

The density distributions of Cα atom distances of Tyr105-Asn132, Lys73-Asn132, Ser70-Gln39, Ser70-Thr269, and residue pairs for all three TEM-1 states are plotted in Figure 11. The Cα atom distance distribution of Tyr105-Asn132 has only one main peak close to 6 Å for reactant state (Figure 11A). However, the conversion from reactant to product leads to a second peak between 8 and 9 Å. Interestingly, the apo state without a ligand shows a similar distance distribution to the product state of this pair with two peaks between 6–7 Å and 8–9 Å. The density distribution of Lys73-Asn132 Cα atom distance has two peaks in the reactant state, one close to 9 Å and one between 10 and 11 Å (Figure 11B). The conversion to the product leads to only one peak around 9.2 Å of this distribution. In apo state, this distribution has a peak around 9.3 Å and a small shoulder about 10.3 Å.

**Figure 12:**
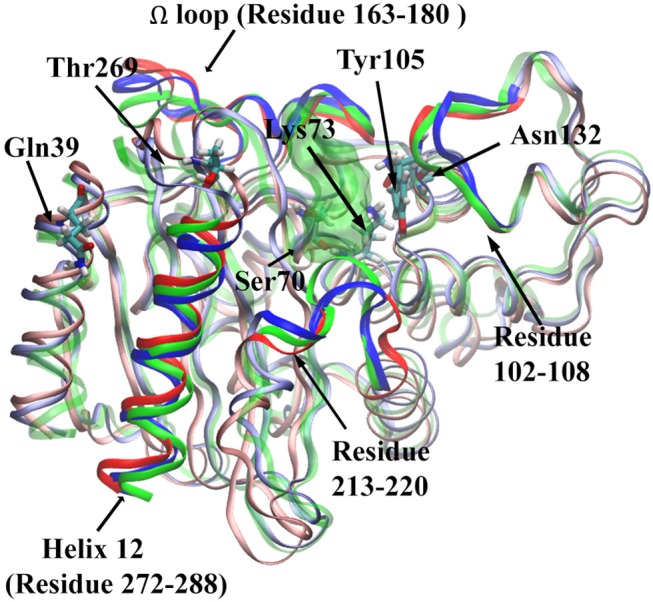
The structure of TEM-1 in apo (blue), reactant (green) and product (red) states. Ω loop (residues 163–180), helix 12 (residues 272–288), residues 102–108, and residues 213–220 are highlighted in each state with same colors. Also key residues Gln39, Thr269, Ser70, Lys73, Tyr105, and Asn132 are labeled. The ligand penicillin-G molecule is represented as green transparent surface.

For Ser70-Gln39 pair, the distributions of their Cα atom distance in all three states have only one peak (Figure 11C), which are located at 23.8, 24, and 24.5 Å for the apo, reactant and product states, respectively. Similarly, the density distributions of Ser70-Thr269 Cα atom distance also have only one peak for all three states, all between 19 and 20 Å (Figure 11D). These analyses demonstrated that the key residues with high feature importance do behave significantly in different functional states of protein. The residues Lys73, Asn132, Gln39, Ser70, and Thr269 are illustrated in the TEM-1 apo, reactant and product aligned structures with green transparent surface representing the ligand penicillin G binding pocket (Figure 12).

**Figure 13:**
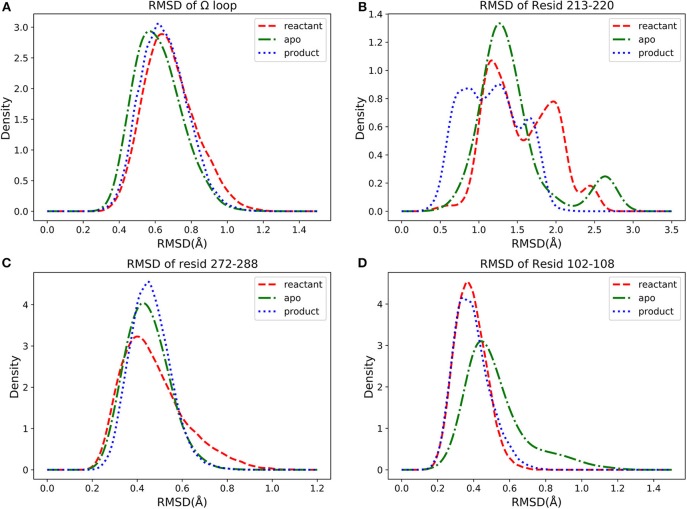
The density distributions of residues groups' RMSDs in apo (green dot dashed line), reactant (red dashed line) and product (blue dot line) states: **(A)** RMSD of Ω loop (residues 163–180), **(B)** RMSD of residues 213–220, **(C)** RMSD of helix 12 (residues 272–288), **(D)** RMSD of residues 102–108.

We further investigated four groups including Ω loop (residues 163–180), residues 213–220 including a turn and random coil structure and residues 102–108 as a turn structure, which are related to structures with high importance percentages illustrated in Figure 7. The helix 12 (residues 272–288) with high importance (>5%) in reactant/product model is also included. To reveal a potentially significant conformational change of these groups, the RMSD of these groups with the TEM-1 (1fqg) crystal structure as a reference are calculated and plotted in Figure 13. In TEM-1 bound with inhibitors, helix 11 (residues 219–226) and helix 12 (residues 272–288) were identified as an allosteric site (Horn and Shoichet, 2004). In the classification models generated in this study, helix 11 has a low feature importance and residues 213–220 have high importance. The RMSD distributions of residues 213–220 and helix 12 as potential allosteric sites are plotted in Figures 13B,C. The RMSD of residues 102–108 as a turn structure containing key residue Tyr105 is plotted in Figure 13D. The positions of the four residues group in TEM-1 are also illustrated in Figure 12. Interestingly, although Ω loop has high importance percentage, the RMSD distributions of Ω loop in three states are similar with each other displaying one main peak around 0.7 Å (Figure 13A). It indicated that Ω loop is not very flexible, agreeing with some NMR studies (Roccatano et al., 2005; Bös and Pleiss, 2009; Fisette et al., 2010). On the contrary, the RMSD distributions of 213–220 turn are significantly different among three states. In the reactant state, there are two main peaks around 1.2 and 2 Å and one small peak around 2.5 Å. In the product state, the RMSD distribution shift toward lower values with three peaks around 0.8, 1.3, and 2.5 Å. In the apo state, there is a dominant peak around 1.3 Å with a smaller peak around 2.6 Å. This clearly revealed significant conformational changes of this turn structure. The RMSD densities of helix 12 (residues 272–288) are similar in all three states with only one peak around 0.4 Å (Figure 13C), suggesting little conformational change of this secondary structure. The RMSD densities of residues 102–108 turn have one dominant distribution in three states (Figure 13D). The reactant and product states have the peak smaller than 0.4 Å. The apo state has the peak larger than 0.4 Å. These analyses demonstrate that the conformational change may play important role only in a limited local structure to differentiate functional states.

## Discussions

The role of protein dynamics in catalysis is becoming essential in understanding enzyme's catalytic mechanisms. TEM-1 is one of the proteins that has been interrogated for the correlation between dynamics and functions both experimentally and computationally (Farmer et al., 2017; Modi and Ozkan, 2018). In a detailed study of TEM-1 using NMR, the backbone motion of several TEM-1 mutants at Tyr105 was characterized and linked to its enzymatic function, because the residue in TEM-1 plays a key role in substrate differentiation and stabilization (Doucet et al., 2007). Coincidently, Tyr105 is identified as the second most important residue to differentiate the apo and product states in the current study (Figure 9A). The NMR study of TEM-1 also revealed that the mutations at residue 105 led to the change of backbone motion exceeding the TEM-1 active site and with a wide range of motion time scales. Interestingly, many key residues discovered in this study to be important for TEM-1 dynamical functional states are in a good agreement with the comprehensive NMR study.

The comparison among NMR spectroscopy of TEM-1 mutants showed that the most significant effect on backbone amide motion, marked as chemical shift differences, occur in the residues in 68–80, 100–115, 120–140, 163–170, 213–218, and 235–246 regions (Doucet et al., 2007). All these regions have significant feature importance from all classification modes developed in the current study (Figure 8). In general, the chemical shift differences observed in NMR spectroscopy have no direct connections with protein dynamics. But the backbone amide chemical shift is sensitive to the hydrogen bonding interactions in protein (Paramasivam et al., 2018). In another study, it was proposed that TEM-1 with mutant Tyr105 displayed effects on the backbone amide chemical shift of wild-type TEM-1 and can reduce the catalytic efficiency of TEM-1 binding with benzyl penicillin complex (Doucet et al., 2004). Although the backbone amide chemical shift difference is caused by the Tyr105 mutation of TEM-1 in the reference, there is indeed a relationship between the chemical shift difference and the catalytic efficiency for TEM-1 with benzyl penicillin complex. Therefore, the correlation between feature importance of key residues with the backbone amide chemical shift differences may help us to further understand the meaning of the machine learning based classification model. It is possible that the backbone amide motion indicated by the NMR spectroscopy is well-coupled with the backbone Cα motion, which is used to construct features for the machine learning training models in this study. Further comparison also shows remarkable agreement at the individual residue level. Some conserved residues and residues at the so-called active site wall showed significant NMR relaxation parameter changes between the wild type and the most significantly different Y105D mutant (Doucet et al., 2007). Six residues (Asn132, Tyr105, Lys215, Val216, Thr71, and Arg243) among the 21 residues with the highest important features from the classification modes in this study (Figure 9) are among the key residues for the local dynamic effects identified in the NMR study. Many more residues (a total of 14) selected by the feature importance are also in the adjacent region within the key residues selected in the NMR study (Table 1).

**Table 1:** The key residues from current study and a NMR study.

**Residues with high feature importance^**a**^**	**Adjacent key NMR residues^**b**^**
Met68, Ser70	Thr71
Ser130, Asp131	Met129 Asn132
Asp163, Trp165, Glu166	Arg164, Glu168
Arg178, Asp179	Thr181
Ala213, Asp214, Ala217	Lys215, Val216, Gly218
Ser235	Lys234
Thr269	Met270

a Current study

b *Table 4 in a NMR study (Doucet et al., 2007)*.

Comparison between the NMR spectroscopy between wild type and Y105D mutant also revealed that significant local differences in the regions of residues 70–80, 124–135, and most importantly in 211–221. Our analysis shows that these regions display high accumulative feature importance as various secondary structures, such as residues 70–80 belonging to α-helix, residues 124–135 spreading across random coil and α-helix, and residues 211–221 containing both random coil and turn structures (Figure 6).

Ω loop (163–180) is a key secondary structure close to the ligand binding site in TEM-1 and important for its catalytic function. A previous NMR and MD simulation work showed that Ω loop displayed limited flexibility with the key translational component (Bös and Pleiss, 2009) It was proposed that the Ω loop is a key structural feature for substrate binding and recognition (Fisette et al., 2012). It was observed in the same study that the inter-Ω loop salt bridge between Arg164 and Asp179 is prone to be affected by the substrate binding, while the Arg164-Thr71 interaction is stabilized by the ligand binding. Accordingly, the Ω loop shows significant and various importance in our three classification models, with the most significance in the non-product/product model. Residues Asp163, Arg164, Trp165, and Asp179 are very important residues (>3% in Figure 8B Ω loop green highlighted part) for the non-product/product differentiation model. Residues Trp165, Glu166, and Glu168 are important residues (>2% in Figure 8B Ω loop green highlighted part) for the apo/product differentiation model. In comparison, the Ω-loop is somewhat less important in the reactant/product model than in the other two models, indicating the importance of differentiating the product from other states. In the non-product/product model, both Arg164 (close to 0.3% percentages of importance) and Asp179 (close to 0.8% percentages of importance) are emphasized as important residues. The Asp179 and Arg164 locate at the entrance of the active site and form the inter-Ω loop salt-bridge to stabilize the loop. In reactant/product and apo/product models, the importance of Arg164 and Asp179 are not obvious, the combination of apo and reactant magnify their importance in non-product/product model. We hypothesized that the interaction between Arg164 and Asp179 exist in all three states to stabilize the loop. Both hydrolyzed benzyl penicillin and benzyl penicillin molecules as substrates can strengthen the interaction. That may be the reason why the overall Ω loop does not carry high importance percentage in reactant/product model. The overall Ω loop is more stable in reactant and product states than in the apo state. In addition, Trp165 is highlighted in both non-product/product and apo/product models, which indicates that Tpr165 is a key residue to classify the apo/reactant and product states. Therefore, it is likely that Tpr165 plays an important role in de-acylation step of the catalytic mechanism, which is also mentioned in experimental study (Petrosino et al., 1998). Another key residue for acylation, Glu166, has a relative high importance in apo/product model. We proposed that Glu166 is not only as a general base in acylation (Minasov et al., 2002) but also very important in the de-acylation step. These detailed comparisons with experimental study provided further insight into the functions of the Ω loop of ligand binding in addition to enzyme catalysis.

The NMR study suggested the key Ω loop motion was in the microsecond (μs)-millisecond (ms) time scale, which was beyond the current simulation study. However, it was also pointed out that the Ω loop dynamics is more focused and less random than other secondary structures even at a large time scale. The good agreement and complimentary comparison between the classification models developed in this study and previous NMR studies of TEM-1 suggests the effectiveness of the machine learning method in the application of protein dynamics and functional analysis. The usage of Cα distance as training features from extensive MD simulations for training practically bridges among protein dynamics with inter-residue correlation, regardless the distance region within the framework of different functional states.

Asn132 was identified as a residue controlling the size of the TEM-1 active site cavity. Distance distribution analysis of Lys73 and Asn132 reveals that the binding with reactant effectively compresses the active site into a closed active site and creates a minor open state representing by two peaks of Cα distance distribution in reactant state (Figure 11B). However, the product binding state only has one main peak as a closed active site without a minor open state. This could be a key dynamical difference between reactant and product binding states. The interaction between Tyr105 and Asn132 also related to the active site. Opposite to the Lys73 and Asn132 Cα distance distribution, the Cα distance distribution of Tyr105 and Asn132 changes from single dominant peak in reactant state to double peaks in the product state (Figure 11A). The difference of the apo state distribution from both reactant and product states also sheds light on these TEM-1 functional states. Helix 11 (residues 219–226) and 12 (residues 272–288) were proposed as an allosteric site with 3–7 Å shift in helix 11 and 1–3 Å shift in helix 12 comparing to the apo structure (Horn and Shoichet, 2004; Avci et al., 2018). The significant conformational change of residues 213–220 as a turn and random coil structure adjacent to helix 11 could be coupled with the allosteric function residing in this region. The similarity of the helix 12 RMSD distributions shared by all three states warrants further study to elucidate the allosteric mechanism associated with this local structure (Figures 13B,C).

It could be a concern that the initial structures for apo, reactant and product state, generated from the same crystal structure (1FQG) in catalytic intermediate state, may not present three target states well. To address this concern, we collected a total of eight crystal structures of wild type TEM-1 in apo states and five crystal structures of wild type TEM-1 binding with various ligands from PDB, including the one with penicillin used as starting structure in this study, as reference structures for the simulations. The averaged RMSDs of each functional state simulations with reference to these crystal structures were calculated and plotted in Supplementary Figure 6. It is interesting that the product state simulations consistently have lower RMSDs with reference to all 13 crystal structures, including both apo and holo states of TEM-1 and the structure used in this study, than both apo and reactant state simulations. In addition, both apo and reactant states simulations consistently have similar RMSDs with reference to these TEM-1 crystal structures. Although these results could prove either the simulations are sufficient for the sampling of each state or not, these results are consistent with our results that the apo and reactant states are similar to each, and both are different from the product state. It may suggest that binding with the catalysis product is a dynamically stable state for TEM-1 and contributes to the catalytic activities of TEM-1 against antibiotics. This could lead to some intrinsic dynamical properties of TEM-1 in different functional states, which warrant further in-depth studies.

## Conclusion

In this study, we developed classification models for TEM-1 β*-*lactamases in different binding modes against penicillin using a machine learning method called random forest. Using the backbone Cα distances of all residue pairs as the features for the model training purpose, the developed classification models effectively correlate the global protein dynamics with the individual residue correlation, with regard to the different binding modes. The feature importance generated from the classification model training process was used to evaluate the contribution from individual residues, as well as secondary structures in TEM-1, to each model. It is shown that the random coil structures carry the highest feature importance among secondary structures, including α-helix, β*-*strands, and turns. It may indicate that the motions of coils contribute significantly to the differences among three states, and lead to more flexibility of random coils than in other secondary structures. Accordingly, the protein flexibility is proposed to be a key factor in ligand recognition of TEM-1. Detailed comparison also revealed that the individual key residues identified from the machine learning models not only have a good agreement with the NMR study, but also provide unprecedented insight into the function of individual residues with regard to differentiating protein in different binding modes. Specifically, it is suggested that some catalytically important residues at the active site are also critical for recognizing the hydrolyzed product of antibiotics. Overall, this study demonstrates that machine learning methods provides effective tools to analyze protein dynamics in different binding modes and produce intriguing insight into the correlation between protein functional states and various structural levels.

## Data Availability

The raw data supporting the conclusions of this manuscript will be made available by the authors, without undue reservation, to any qualified researcher.

## Author Contributions

FW wrote the manuscript and carried out the four independent MD simulations for three states (1,200 ns) and performed all the analysis. LS carried out 1 MD simulation for three states (300 ns). HZ provided some scripts of machine learning. SW and XW authors contributed to the final version of the manuscript. PT contributed to the final version of the manuscript and supervised the project.

### Conflict of Interest Statement

The authors declare that the research was conducted in the absence of any commercial or financial relationships that could be construed as a potential conflict of interest.
