# Supplementary material for: Machine Learning Classification Model for Functional Binding Modes of TEM-1 β-Lactamase
Source: Front Mol Biosci. 2019 Jul 9;6:47. doi: 10.3389/fmolb.2019.00047 (PMC6629954; doi:10.3389/fmolb.2019.00047)
Supplement: Supplementary file 1 [file Data_Sheet_1.PDF]

Supplementary Material

1 Supplementary Data

1.1 The preselected features of apo/product model

The numbers of preselected features (901) of apo/product model is listed below as the format [%d1-%d2], %d1 and %d2 are the residue numbers of TEM-1. They are the pairwise residues on TEM-1 used for apo/product training model in Random Forest.

[210-214], [72-164], [213-286], [68-246], [68-260], [213-265], [168-236], [182-213], [213-278], [164-208], [130-258], [164-247], [63-213], [105-170], [171-174], [88-213], [178-216], [179-232], [199-214], [165-217], [130-172], [74-163], [42-213], [215-233], [167-236], [217-240], [214-218], [211-242], [74-246], [70-130], [83-217], [52-215], [210-216], [104-167], [219-233], [210-213], [213-240], [72-280], [171-216], [131-230], [80-213], [173-217], [179-222], [26-166], [119-218], [96-213], [259-266], [74-130], [170-217], [170-236], [256-266], [213-244], [68-139], [57-172], [172-258], [130-244], [47-266], [215-242], [121-214], [169-205], [60-105], [134-231], [213-235], [166-247], [72-165], [165-245], [251-266], [214-219], [83-213], [119-217], [70-232], [75-163], [73-165], [131-168], [215-260], [223-248], [49-104], [109-231], [26-94], [75-241], [68-279], [172-251], [76-219], [30-213], [26-60], [213-273], [68-262], [213-283], [105-196], [213-232], [26-187], [26-147], [120-212], [172-256], [44-212], [208-266], [154-217], [217-288], [104-189], [172-224], [213-274], [166-234], [187-217], [209-219], [83-135], [172-247], [40-213], [212-214], [68-222], [109-222], [147-225], [208-217], [104-146], [106-165], [130-238], [61-177], [166-231], [103-222], [68-164], [216-248], [105-159], [58-68], [187-219], [68-273], [187-266], [262-277], [26-237], [62-202], [38-105], [120-234], [170-173], [154-218], [176-238], [70-167], [213-279], [91-221], [41-213], [169-234], [171-173], [215-223], [72-217], [45-219], [49-129], [70-140], [73-130], [96-222], [172-260], [90-212], [214-233], [213-243], [70-166], [213-219], [46-197], [168-248], [213-263], [217-239], [44-214], [107-163], [44-68], [46-105], [47-130], [105-198], [129-218], [118-213], [95-222], [83-214], [164-215], [34-74], [70-246], [62-71], [205-219], [144-217], [56-63], [75-173], [97-222], [164-245], [107-153], [33-68], [133-231], [40-215], [30-75], [142-218], [255-266], [42-217], [215-279], [119-239], [213-261], [165-261], [70-204], [201-217], [130-260], [51-197], [150-178], [215-264], [37-218], [68-216], [215-230], [164-222], [217-241], [222-278], [60-106], [52-130], [172-217], [46-123], [127-240], [236-263], [118-212], [72-276], [62-249], [218-275], [68-271], [172-187], [173-204], [165-235], [102-234], [82-240], [48-213], [26-120], [136-234], [73-132], [26-161], [41-218], [159-223], [36-218], [54-214], [70-169], [86-284], [197-257], [130-179], [76-186], [49-221], [57-236], [127-224], [104-264], [117-213], [51-216], [213-285], [98-230], [212-219], [26-107], [48-197], [70-170], [216-270], [74-218], [86-213], [132-219], [63-247], [70-208], [215-277], [105-158], [105-263], [163-222], [91-213], [212-215], [143-220], [178-258], [55-130], [210-218], [104-239], [213-276], [119-213], [129-166], [105-239], [177-230], [177-217], [68-263], [216-218], [119-136], [179-261], [78-179], [63-230], [139-216], [182-215], [63-277], [219-288], [164-236], [132-234], [33-128], [31-272], [30-68], [103-247], [197-218], [244-277], [131-247], [266-285], [26-151], [44-213], [67-80], [131-257], [83-101], [33-130], [93-213], [83-169], [216-241], [105-135], [107-161], [222-286], [104-174], [118-282], [168-250], [129-217], [37-68], [185-219], [26-247], [81-213], [42-197], [217-253], [47-223], [105-220], [218-232], [70-257], [167-247], [166-213], [77-106], [165-214], [215-248], [214-269], [71-166], [76-223], [166-259], [130-239], [49-236], [27-35], [207-224], [60-197], [223-230], [49-197], [242-266], [45-213], [220-261], [63-266], [216-249], [26-

201], [167-243], [215-240], [81-182], [48-221], [116-220], [95-213], [67-215], [68-144], [165-222], [82-104], [213-288], [222-255], [172-265], [216-265], [26-72], [136-236], [44-164], [213-234], [197-286], [29-130], [235-266], [43-213], [106-158], [172-189], [43-228], [201-214], [75-148], [70-132], [130-162], [134-215], [82-92], [261-275], [218-243], [106-219], [64-280], [130-254], [168-231], [63-155], [163-262], [108-197], [213-231], [71-133], [224-242], [33-129], [164-246], [104-281], [80-214], [218-224], [99-215], [105-140], [60-215], [63-73], [126-224], [105-260], [70-101], [152-217], [26-71], [230-266], [62-190], [107-184], [214-275], [122-213], [172-248], [224-249], [140-223], [121-263], [198-219], [68-217], [217-222], [51-113], [166-187], [172-234], [52-221], [215-245], [126-222], [197-284], [58-198], [206-218], [38-195], [166-229], [115-216], [162-164], [127-220], [214-274], [162-246], [122-219], [212-239], [68-245], [130-189], [72-131], [55-64], [132-168], [172-228], [47-217], [203-218], [169-215], [216-247], [127-223], [218-276], [26-79], [43-218], [26-157], [26-236], [148-219], [127-234], [67-76], [176-240], [104-201], [77-219], [44-106], [94-219], [132-215], [181-215], [47-105], [184-221], [211-247], [137-216], [86-107], [121-218], [26-191], [74-265], [214-261], [104-177], [183-197], [73-135], [128-232], [134-216], [193-202], [210-215], [63-223], [117-133], [143-217], [164-224], [104-168], [105-181], [86-214], [77-268], [218-244], [30-193], [188-236], [77-222], [95-220], [214-244], [66-117], [104-178], [43-100], [81-190], [179-262], [50-221], [63-122], [30-199], [71-173], [68-73], [93-137], [121-213], [56-70], [217-260], [26-109], [215-250], [86-218], [26-131], [130-259], [30-192], [135-236], [238-278], [64-161], [165-231], [99-222], [70-280], [105-153], [108-166], [70-149], [26-106], [171-222], [122-218], [87-248], [188-199], [266-288], [50-216], [63-255], [106-164], [63-87], [105-177], [42-287], [33-133], [165-246], [26-84], [104-261], [30-127], [72-162], [96-210], [105-266], [26-50], [217-261], [169-237], [54-91], [118-141], [72-132], [26-242], [168-244], [70-106], [140-161], [46-163], [43-85], [26-55], [108-243], [85-235], [149-261], [222-232], [223-253], [28-235], [105-199], [70-248], [130-186], [30-167], [103-224], [135-183], [34-179], [62-236], [29-129], [26-269], [217-244], [114-216], [142-154], [26-148], [220-258], [105-210], [260-280], [171-236], [74-165], [59-221], [213-275], [65-236], [261-266], [44-177], [214-221], [245-282], [42-62], [38-207], [138-182], [81-223], [192-206], [213-266], [74-224], [70-168], [27-222], [213-221], [31-146], [27-52], [104-275], [130-170], [147-174], [26-232], [30-165], [100-235], [73-131], [67-186], [217-278], [166-262], [105-185], [43-68], [32-220], [107-168], [87-197], [133-223], [214-237], [186-266], [213-236], [87-153], [211-235], [29-115], [43-170], [71-164], [133-214], [26-170], [30-234], [91-222], [27-30], [63-104], [104-247], [216-245], [87-288], [105-152], [39-219], [116-280], [256-270], [26-256], [111-136], [165-262], [30-232], [26-164], [131-179], [39-104], [112-122], [207-213], [131-139], [72-250], [216-254], [217-259], [128-218], [104-149], [45-203], [86-216], [216-237], [51-266], [76-104], [67-107], [107-213], [207-218], [117-136], [48-105], [105-157], [178-211], [84-233], [120-262], [62-89], [104-173], [90-277], [45-68], [102-232], [112-165], [248-255], [26-101], [146-215], [75-119], [160-194], [130-191], [75-131], [71-132], [107-273], [44-105], [105-131], [112-188], [33-123], [185-211], [26-110], [185-217], [111-268], [35-71], [220-264], [172-249], [212-234], [132-250], [77-105], [71-105], [26-193], [87-182], [200-236], [117-150], [155-275], [74-196], [84-255], [74-223], [70-165], [27-282], [74-262], [62-117], [116-250], [139-271], [94-213], [72-109], [247-262], [217-231], [46-104], [122-187], [176-218], [213-245], [183-220], [211-217], [125-197], [47-61], [125-247], [64-76], [47-236], [218-247], [213-272], [30-211], [42-117], [61-216], [103-236], [105-223], [108-133], [95-243], [103-282], [51-215], [152-281], [106-134], [233-283], [224-285], [60-81], [73-189], [44-165], [26-87], [250-271], [30-130], [172-227], [33-168], [164-197], [46-177], [26-59], [93-109], [161-219], [119-268], [141-244], [56-197], [90-247], [205-217], [183-282], [122-261], [139-231], [217-275], [61-214], [170-225], [27-231], [93-247], [123-245], [71-167], [58-205], [104-263], [246-281], [26-212], [179-216], [73-237], [173-218], [106-205], [138-232], [174-222], [71-197], [40-263], [220-252], [43-216], [221-249], [56-129], [145-239], [156-255], [170-263], [78-104], [109-160], [28-213], [91-174], [104-132], [77-188], [146-196], [57-200], [53-241], [150-283], [130-163], [70-

137], [64-191], [26-139], [90-246], [82-199], [166-236], [142-175], [37-67], [156-259], [54-220], [88-105], [89-222], [138-198], [106-166], [61-222], [123-238], [179-195], [92-214], [47-177], [70-220], [60-272], [115-213], [32-213], [86-106], [163-225], [60-68], [62-134], [39-132], [218-270], [212-263], [223-233], [63-279], [82-94], [71-129], [27-211], [161-245], [218-233], [168-209], [131-222], [187-204], [130-142], [134-177], [69-81], [90-210], [69-92], [33-76], [213-224], [36-216], [104-109], [29-76], [34-116], [242-272], [49-72], [189-234], [174-200], [216-263], [184-236], [178-235], [110-187], [178-215], [75-139], [73-146], [49-222], [129-225], [70-237], [130-164], [213-230], [104-235], [109-139], [245-281], [213-282], [103-219], [160-224], [124-218], [30-126], [39-262], [175-285], [154-194], [34-118], [64-158], [168-263], [102-120], [65-268], [30-219], [204-223], [37-76], [100-196], [192-199], [68-261], [208-234], [212-217], [63-285], [36-236], [62-207], [212-245], [88-206], [239-274], [131-161], [61-68], [224-273], [107-268], [86-228], [143-179], [56-170], [42-90], [93-218], [83-183], [131-262], [72-136], [106-148], [113-115], [151-235], [174-227], [149-215], [219-285], [43-142], [108-180], [130-231], [48-267], [132-159], [214-249]

1.2 The preselected features of reactant/product model

The numbers of preselected features (1170) of reactant/product model is listed below as the format [%d1-%d2], %d1 and %d2 are the residue numbers of TEM1. They are the pairwise residues on TEM-1 used for reactant/product training model in Random Forest.

[215-261], [215-218], [69-179], [213-278], [70-191], [214-245], [70-143], [70-205], [108-213], [69-242], [65-166], [90-213], [164-272], [163-269], [147-269], [58-214], [179-274], [112-215], [213-233], [129-272], [73-144], [109-274], [62-165], [137-238], [213-265], [79-170], [147-167], [70-230], [70-232], [48-213], [105-139], [169-270], [70-259], [46-215], [213-258], [122-216], [67-166], [71-170], [70-249], [164-268], [71-171], [70-168], [213-277], [182-213], [43-129], [117-216], [70-162], [103-216], [72-274], [214-235], [44-147], [117-215], [218-272], [46-148], [163-243], [182-215], [69-236], [32-215], [70-145], [146-262], [120-218], [45-129], [70-165], [215-280], [82-270], [71-132], [213-272], [119-216], [179-271], [83-214], [132-217], [71-165], [32-270], [61-149], [70-258], [45-212], [73-149], [65-213], [64-229], [264-266], [35-213], [69-264], [130-261], [60-147], [130-237], [130-283], [109-271], [214-273], [263-266], [68-105], [160-246], [212-214], [69-177], [129-216], [70-131], [210-213], [158-166], [27-31], [166-231], [165-182], [169-214], [65-176], [43-146], [26-33], [106-130], [27-148], [26-271], [179-228], [71-116], [26-104], [73-269], [148-216], [125-214], [196-249], [30-268], [243-274], [125-224], [68-132], [216-218], [66-166], [237-271], [70-214], [47-68], [44-216], [81-179], [77-218], [190-266], [69-269], [35-275], [66-132], [83-238], [27-265], [209-266], [118-271], [26-164], [62-120], [70-73], [213-218], [121-216], [40-237], [104-131], [70-231], [149-243], [215-247], [142-218], [104-189], [71-145], [128-214], [31-216], [82-91], [210-214], [140-167], [72-266], [62-266], [50-213], [69-129], [214-221], [133-166], [165-257], [130-178], [213-283], [48-72], [90-223], [31-219], [71-163], [165-266], [71-100], [217-248], [148-272], [130-190], [63-202], [148-242], [105-264], [178-231], [72-271], [69-130], [119-213], [99-105], [165-230], [135-269], [118-239], [69-77], [213-244], [63-284], [140-165], [211-218], [82-106], [164-178], [67-73], [163-270], [126-259], [209-213], [72-105], [63-166], [148-219], [71-143], [32-213], [91-217], [137-219], [215-260], [163-242], [81-122], [132-215], [216-250], [219-267], [105-154], [194-233], [129-166], [145-214], [28-165], [235-243], [149-273], [180-214], [70-235], [211-215], [138-198], [136-270], [95-218], [52-165], [69-170], [144-270], [50-197], [173-187], [191-217], [212-259], [71-148], [84-269], [40-64], [126-215], [125-240], [111-217], [237-266], [145-276], [36-84], [183-215], [107-274], [26-219], [215-220], [71-258], [165-220], [36-216], [26-169], [204-214], [215-254], [26-212], [51-87], [62-71], [26-139], [83-218], [83-142], [146-221], [214-217], [89-158], [215-255], [27-180], [221-266], [77-271], [28-219], [163-177], [159-244], [32-175], [69-74], [137-239], [160-213], [139-167], [32-269], [26-153], [165-180], [102-165], [65-214], [67-124], [104-248], [85-257], [213-260], [215-258], [214-222], [131-

197], [27-267], [86-211], [70-215], [68-179], [64-211], [61-213], [26-249], [58-118], [101-264], [144-267], [178-214], [168-222], [107-165], [144-259], [57-218], [26-163], [130-280], [131-229], [144-269], [80-104], [149-235], [68-170], [220-245], [64-248], [130-262], [266-285], [197-235], [26-65], [196-237], [26-55], [215-259], [26-43], [215-281], [145-274], [180-219], [140-198], [61-166], [62-84], [43-242], [166-247], [104-281], [63-231], [143-270], [147-241], [70-240], [216-287], [72-162], [166-214], [29-165], [47-70], [26-74], [71-139], [77-169], [26-96], [210-271], [125-178], [166-237], [218-287], [71-179], [65-244], [96-215], [94-212], [116-215], [165-256], [217-252], [46-244], [89-222], [90-220], [39-274], [27-36], [46-213], [77-128], [214-266], [124-270], [51-196], [236-275], [164-231], [164-270], [203-217], [26-162], [88-104], [170-270], [104-201], [236-249], [38-269], [120-238], [144-178], [46-68], [195-231], [29-70], [67-165], [72-169], [38-144], [160-234], [162-236], [260-267], [39-275], [188-217], [37-269], [236-274], [109-218], [143-168], [26-79], [64-193], [131-221], [27-175], [213-286], [128-266], [68-121], [81-277], [64-234], [105-125], [143-165], [64-183], [67-122], [127-271], [126-230], [43-236], [179-211], [26-59], [72-270], [141-173], [147-183], [101-270], [26-281], [144-179], [218-244], [104-270], [62-246], [104-283], [159-217], [40-76], [150-185], [70-167], [26-66], [62-74], [70-250], [102-185], [31-192], [90-214], [63-71], [107-176], [121-141], [79-239], [40-70], [95-213], [86-274], [94-214], [104-199], [107-175], [134-274], [161-244], [26-187], [218-224], [217-288], [147-209], [236-270], [79-266], [135-239], [217-261], [69-245], [98-216], [71-150], [217-233], [86-231], [127-247], [74-274], [97-103], [104-155], [244-271], [218-277], [150-165], [63-185], [62-188], [32-284], [30-219], [39-214], [77-270], [196-248], [26-143], [62-250], [174-216], [172-261], [36-104], [126-219], [79-195], [70-169], [75-147], [27-149], [39-272], [31-114], [174-272], [110-219], [109-241], [80-242], [70-248], [77-178], [129-164], [27-208], [106-215], [76-147], [47-73], [30-272], [211-274], [216-265], [214-260], [34-196], [164-237], [110-167], [104-132], [146-170], [39-271], [100-216], [86-225], [49-70], [66-104], [30-47], [37-281], [62-81], [26-265], [45-67], [140-270], [70-186], [94-209], [105-159], [170-271], [108-220], [70-213], [133-176], [63-119], [218-253], [215-226], [137-270], [127-178], [107-145], [69-187], [90-232], [216-282], [33-146], [31-276], [70-164], [171-271], [144-262], [131-239], [114-216], [64-244], [81-166], [179-215], [79-104], [26-216], [57-216], [86-194], [63-121], [213-249], [163-268], [88-195], [134-216], [214-238], [136-216], [46-167], [26-102], [139-158], [40-73], [81-133], [148-214], [27-60], [243-273], [130-165], [63-74], [141-217], [136-240], [145-283], [204-254], [35-271], [67-111], [178-271], [28-79], [27-184], [167-271], [213-242], [162-214], [63-214], [47-214], [245-274], [86-197], [61-176], [64-257], [117-158], [132-166], [148-269], [39-64], [83-164], [47-220], [69-280], [106-199], [236-248], [53-215], [43-220], [53-218], [63-126], [49-267], [179-226], [159-192], [32-209], [131-189], [107-164], [131-179], [71-169], [98-239], [94-216], [76-271], [143-269], [97-275], [63-80], [218-233], [65-177], [124-269], [215-284], [267-282], [64-266], [136-237], [37-272], [81-104], [213-261], [128-213], [144-225], [229-240], [149-215], [70-128], [213-224], [43-239], [219-265], [38-266], [219-279], [219-277], [132-258], [39-169], [178-225], [142-156], [217-231], [30-190], [136-251], [57-65], [46-105], [78-215], [63-104], [72-131], [54-104], [213-217], [146-270], [124-213], [43-123], [43-170], [121-218], [215-240], [180-231], [179-232], [44-217], [36-269], [86-250], [65-173], [136-269], [195-245], [104-159], [32-146], [62-130], [164-269], [42-129], [34-219], [63-105], [69-145], [145-220], [26-172], [126-269], [218-264], [110-271], [45-82], [145-273], [122-223], [94-213], [157-193], [143-259], [152-178], [68-217], [191-243], [93-144], [247-279], [168-237], [64-237], [244-269], [72-269], [32-176], [206-270], [139-229], [114-261], [125-168], [180-270], [106-186], [84-102], [60-178], [147-237], [148-263], [139-166], [77-174], [40-143], [82-125], [81-134], [104-126], [169-269], [96-159], [91-143], [111-268], [215-231], [88-270], [219-247], [131-178], [181-266], [105-238], [63-186], [226-245], [61-288], [64-98], [86-136], [40-69], [217-259], [190-217], [73-163], [213-225], [215-251], [188-221], [167-212], [146-271], [30-237], [152-271], [83-123], [45-214], [219-236], [152-283], [147-259], [105-269], [27-164], [214-236], [128-270], [86-218], [215-239], [30-275], [130-236], [52-87], [178-182], [34-85], [102-190], [191-

206], [26-258], [127-134], [71-166], [143-241], [39-86], [73-118], [136-178], [227-280], [93-159], [169-193], [163-214], [194-249], [71-135], [218-278], [160-266], [121-189], [118-135], [53-267], [82-217], [27-283], [26-210], [135-260], [69-229], [102-217], [83-105], [120-217], [48-197], [50-71], [26-256], [88-198], [165-272], [139-272], [27-163], [106-118], [190-248], [129-168], [71-233], [63-233], [165-184], [198-233], [26-224], [111-166], [64-235], [38-279], [183-268], [37-215], [162-200], [107-157], [181-208], [66-284], [102-173], [63-272], [62-93], [132-222], [145-178], [104-245], [61-89], [72-164], [162-209], [67-133], [70-239], [74-239], [28-271], [131-274], [105-170], [175-182], [104-127], [224-244], [58-217], [26-201], [51-276], [257-266], [54-105], [104-152], [85-99], [43-61], [196-205], [123-270], [27-221], [105-186], [106-124], [171-214], [110-229], [112-218], [116-271], [218-234], [109-213], [71-144], [28-217], [234-270], [119-198], [283-288], [143-224], [147-214], [43-144], [156-180], [149-270], [84-220], [123-178], [222-261], [71-134], [26-254], [100-222], [217-280], [29-46], [67-200], [32-239], [141-161], [81-128], [143-170], [78-217], [50-217], [151-200], [226-244], [132-235], [108-141], [71-191], [72-138], [93-152], [59-220], [72-168], [104-185], [186-219], [27-179], [64-104], [79-218], [124-268], [166-201], [198-265], [163-220], [128-274], [35-87], [73-271], [136-187], [190-218], [63-136], [63-169], [135-158], [27-223], [114-221], [70-151], [42-68], [108-269], [217-265], [88-170], [169-257], [64-227], [144-247], [178-247], [163-262], [62-187], [70-245], [75-272], [209-217], [37-104], [33-43], [38-150], [166-271], [72-146], [102-267], [79-132], [70-163], [121-223], [224-250], [205-213], [26-91], [28-146], [124-168], [36-82], [93-157], [196-217], [150-220], [163-222], [81-261], [190-222], [150-261], [105-247], [110-251], [70-242], [61-150], [215-274], [67-231], [236-247], [63-78], [244-251], [139-216], [70-178], [63-249], [146-243], [117-133], [27-154], [80-218], [43-103], [212-261], [151-238], [100-277], [212-215], [69-176], [193-202], [161-214], [86-202], [71-110], [160-186], [42-235], [33-45], [35-124], [40-202], [259-270], [98-199], [119-275], [105-113], [190-209], [126-197], [41-83], [91-103], [146-161], [49-80], [72-136], [88-143], [193-216], [97-216], [74-269], [61-66], [108-127], [201-218], [60-165], [91-179], [43-243], [150-170], [88-271], [69-210], [68-108], [147-226], [50-142], [170-245], [108-270], [31-277], [26-182], [146-246], [138-197], [178-287], [85-120], [62-285], [70-233], [60-82], [106-219], [71-288], [168-231], [224-252], [60-72], [61-105], [70-122], [266-278], [90-268], [111-192], [75-205], [216-221], [90-97], [230-244], [40-81], [57-66], [57-215], [216-229], [26-193], [179-208], [63-106], [63-228], [105-171], [43-209], [141-149], [63-132], [68-270], [59-284], [74-224], [55-159], [62-86], [222-244], [180-249], [169-216], [40-217], [101-266], [176-184], [150-274], [81-105], [204-283], [71-235], [151-252], [68-260], [161-195], [40-148], [137-218], [34-67], [66-135], [86-219], [220-284], [106-192], [171-209], [128-237], [30-62], [45-61], [92-219], [124-218], [66-105], [263-274], [31-93], [149-233], [59-67], [58-220], [64-232], [94-171], [201-237], [26-247], [263-271], [90-93], [77-274], [29-159], [44-145], [93-177], [116-230], [189-221], [45-150], [181-239], [77-202], [67-168], [52-220], [67-104], [135-285], [27-54], [53-171], [62-92], [91-115], [195-217], [108-129], [28-63], [133-255], [170-230], [39-133], [168-271], [213-232], [126-140], [103-164], [169-267], [121-180], [36-105], [215-236], [207-284], [104-192], [38-153], [35-122], [46-133], [82-208], [75-214], [234-236], [62-131], [219-285], [63-178], [69-209], [239-271], [213-256], [146-222], [46-130], [70-146], [139-266], [54-209], [61-175], [213-268], [213-266], [43-50], [125-262], [178-237], [59-218], [111-127], [30-279], [128-179], [61-73], [106-270], [195-204], [70-257], [129-192], [134-209], [27-242], [38-224], [217-270], [93-178], [224-266], [91-275], [217-266], [63-138], [45-280], [46-219], [50-159], [31-271], [96-270], [37-195], [69-178], [31-282], [101-269], [44-131], [164-271], [69-148], [26-184], [167-210], [88-237], [59-276], [37-45], [255-287], [194-229], [151-203], [221-268], [26-35], [154-161], [56-218], [164-179], [155-278], [233-271], [26-147], [137-172], [120-178], [136-172], [192-241], [63-190], [194-258], [63-176], [111-153], [27-64], [34-73], [217-268], [184-267], [148-261], [257-263], [84-129], [35-204], [26-226], [72-141], [219-271], [35-196], [197-257], [147-149], [157-262], [139-241], [221-244]

1.3 The preselected features of non-product/product model

The numbers of preselected features of non-product/product (964) model is listed below as the format [%d1-%d2], %d1 and %d2 are the residue numbers of TEM1. They are the pairwise residues on TEM-1 used for non-product/product training model in Random Forest.

[68-262], [70-168], [71-168], [70-169], [46-68], [68-270], [163-217], [68-245], [70-101], [71-185], [130-235], [44-129], [164-216], [129-268], [130-241], [128-276], [130-262], [164-234], [215-258], [94-237], [55-215], [164-223], [70-249], [82-271], [213-229], [214-283], [71-235], [95-213], [82-270], [214-219], [213-266], [144-217], [69-170], [90-213], [208-219], [71-126], [135-270], [81-274], [26-59], [140-271], [61-68], [214-279], [60-130], [121-214], [49-266], [203-219], [119-220], [84-170], [172-224], [146-215], [79-222], [170-245], [107-224], [171-216], [68-220], [65-165], [177-217], [164-250], [75-219], [119-222], [26-159], [44-213], [214-218], [67-92], [168-217], [128-269], [124-272], [145-217], [72-271], [153-234], [117-216], [124-213], [134-274], [39-213], [140-169], [72-242], [130-261], [207-274], [74-130], [26-48], [103-233], [168-246], [216-232], [116-216], [126-233], [143-270], [64-237], [69-213], [148-216], [171-214], [72-270], [39-214], [78-178], [125-218], [129-237], [131-247], [72-274], [189-218], [130-237], [26-64], [69-119], [70-163], [73-164], [129-275], [124-269], [27-215], [179-202], [71-102], [136-165], [74-166], [27-161], [87-187], [165-245], [217-239], [236-266], [117-213], [120-219], [217-232], [68-243], [70-188], [191-197], [213-241], [109-216], [182-213], [217-259], [132-215], [82-213], [26-180], [26-168], [164-217], [70-240], [50-215], [26-281], [213-273], [71-242], [149-268], [60-68], [150-182], [26-170], [213-272], [26-104], [31-70], [165-216], [179-271], [116-215], [60-185], [69-171], [59-197], [60-145], [107-238], [168-251], [105-150], [213-288], [66-166], [125-266], [89-169], [138-217], [193-219], [129-222], [125-214], [219-244], [80-239], [132-161], [128-218], [71-271], [145-233], [36-217], [167-224], [218-271], [213-242], [216-223], [68-166], [169-231], [66-165], [60-66], [27-285], [26-125], [72-241], [70-167], [70-131], [126-170], [32-275], [46-179], [44-214], [26-66], [252-274], [26-140], [213-220], [107-163], [127-164], [165-251], [109-286], [64-260], [64-244], [203-214], [71-130], [26-187], [137-215], [43-72], [93-213], [216-225], [105-158], [133-243], [163-247], [213-238], [178-243], [91-166], [238-249], [77-271], [217-265], [63-72], [43-64], [217-234], [144-170], [219-245], [104-149], [180-260], [104-184], [237-266], [43-59], [26-259], [48-105], [208-218], [187-266], [104-129], [72-285], [149-264], [26-76], [62-275], [123-271], [47-218], [70-255], [104-268], [27-216], [181-262], [210-218], [26-284], [120-213], [93-105], [162-216], [114-213], [100-215], [209-271], [217-242], [60-162], [165-260], [127-245], [26-151], [26-172], [213-235], [26-149], [33-68], [218-276], [144-176], [43-130], [140-216], [237-274], [216-218], [70-217], [87-214], [35-275], [55-266], [108-125], [70-134], [40-165], [218-269], [130-170], [26-211], [242-266], [109-166], [131-240], [130-246], [31-69], [138-218], [34-105], [169-217], [126-269], [43-67], [210-238], [132-169], [131-235], [52-197], [70-232], [211-214], [135-238], [121-218], [152-271], [130-187], [149-210], [89-141], [27-224], [165-285], [36-63], [58-213], [70-166], [62-261], [86-106], [106-137], [68-143], [144-197], [71-264], [131-215], [74-242], [261-266], [139-270], [81-218], [129-164], [63-216], [73-131], [104-243], [230-236], [26-252], [166-233], [133-233], [26-260], [108-137], [45-149], [130-249], [31-72], [178-270], [71-171], [197-245], [26-148], [62-259], [26-42], [70-211], [170-274], [31-278], [27-41], [107-258], [191-266], [115-268], [107-268], [91-269], [27-265], [166-230], [42-197], [166-223], [66-274], [71-191], [27-268], [104-135], [41-105], [177-182], [179-260], [186-196], [64-282], [71-166], [164-271], [128-214], [32-246], [171-226], [166-217], [26-154], [32-165], [163-263], [218-248], [135-168], [83-216], [62-79], [63-119], [34-147], [220-265], [68-107], [100-224], [42-109], [64-239], [32-161], [214-217], [63-210], [26-144], [129-270], [71-137], [206-214], [173-213], [197-218], [40-279], [74-274], [129-235], [40-213], [150-220], [154-217], [68-215], [82-162], [162-202], [105-265], [26-82], [105-193], [47-104], [130-234], [165-250], [165-279], [81-

214], [197-262], [63-132], [50-105], [77-105], [67-213], [63-124], [178-199], [215-257], [183-220],
[215-218], [180-228], [188-216], [210-270], [105-285], [27-179], [97-216], [105-251], [91-274], [44-
166], [165-242], [104-288], [63-187], [108-126], [27-43], [104-241], [55-217], [68-230], [26-254],
[98-188], [232-266], [33-130], [70-268], [100-168], [248-273], [26-201], [150-261], [107-167], [75-
165], [65-244], [150-271], [100-104], [72-262], [190-217], [116-237], [35-93], [106-141], [38-214],
[61-150], [70-75], [83-217], [197-250], [88-240], [216-284], [84-243], [68-235], [215-229], [105-278],
[71-205], [68-214], [96-167], [26-98], [35-269], [78-271], [31-178], [166-244], [179-207], [85-229],
[180-246], [166-283], [131-228], [28-104], [27-278], [72-263], [129-165], [218-265], [130-272], [197-
275], [215-270], [217-241], [37-146], [26-133], [218-233], [244-270], [179-238], [105-135], [144-
177], [166-220], [68-116], [104-221], [147-251], [44-145], [89-270], [29-102], [165-236], [62-72],
[194-219], [43-68], [26-280], [26-212], [104-258], [59-81], [181-244], [68-165], [129-141], [58-287],
[43-265], [119-263], [104-198], [111-269], [218-230], [163-234], [117-143], [72-243], [197-272],
[185-205], [132-166], [161-274], [253-266], [30-99], [30-187], [61-220], [216-230], [26-181], [164-
224], [178-271], [35-69], [149-222], [146-224], [128-253], [199-217], [42-228], [49-130], [71-219],
[213-262], [106-224], [109-275], [165-181], [70-128], [98-237], [26-115], [26-84], [89-178], [178-
241], [83-212], [102-107], [210-213], [44-149], [86-184], [150-241], [220-278], [219-271], [79-270],
[234-285], [89-219], [65-237], [40-70], [104-222], [94-213], [125-213], [39-130], [170-224], [215-
267], [170-271], [64-151], [126-257], [72-221], [68-217], [47-214], [125-216], [148-208], [217-254],
[74-271], [214-269], [89-239], [41-265], [28-105], [198-280], [130-274], [38-104], [258-266], [126-
246], [66-266], [60-163], [37-219], [100-261], [262-274], [72-163], [163-270], [103-190], [217-247],
[156-271], [79-183], [91-210], [105-130], [70-113], [214-248], [74-224], [77-106], [66-106], [58-182],
[102-217], [68-90], [215-284], [28-47], [44-133], [93-217], [63-254], [80-277], [35-138], [156-194],
[213-217], [254-266], [63-197], [62-147], [185-221], [207-213], [26-107], [215-225], [50-216], [60-
213], [117-247], [81-189], [61-197], [98-273], [28-288], [76-148], [67-270], [107-269], [244-275],
[165-235], [144-261], [88-222], [43-47], [37-147], [69-78], [130-282], [106-139], [50-86], [31-236],
[92-220], [62-251], [119-271], [130-233], [44-73], [90-215], [70-108], [34-275], [157-281], [130-245],
[81-238], [40-43], [197-264], [192-196], [71-220], [105-176], [74-145], [117-186], [91-271], [96-222],
[213-275], [172-225], [105-170], [71-217], [126-181], [167-209], [215-221], [46-131], [72-165], [35-
271], [236-274], [209-215], [131-262], [159-261], [74-270], [107-267], [130-224], [67-104], [180-
219], [64-145], [86-203], [195-217], [216-238], [133-215], [70-252], [113-166], [170-236], [265-288],
[134-178], [85-227], [46-284], [44-108], [154-283], [201-218], [63-275], [245-266], [105-272], [64-
256], [145-270], [64-162], [213-239], [178-274], [61-66], [66-212], [219-267], [82-179], [38-102],
[105-120], [49-263], [179-283], [40-138], [104-123], [149-216], [104-283], [128-161], [26-262], [52-
104], [132-149], [185-266], [123-217], [177-261], [129-173], [70-214], [103-216], [215-253], [189-
266], [43-121], [47-131], [71-129], [148-217], [212-217], [56-105], [216-261], [40-170], [72-237],
[179-270], [29-208], [124-262], [76-196], [113-270], [109-174], [109-118], [93-218], [125-167], [80-
159], [62-279], [218-239], [71-237], [74-179], [136-217], [104-182], [133-145], [212-214], [28-145],
[167-217], [122-261], [79-214], [26-130], [36-64], [163-174], [260-269], [201-210], [187-288], [61-
158], [67-83], [162-275], [213-222], [212-220], [183-229], [101-235], [31-83], [215-230], [195-229],
[49-93], [139-256], [129-282], [142-166], [153-182], [76-240], [179-216], [127-139], [204-262], [27-
38], [180-231], [84-180], [32-132], [109-117], [210-263], [86-136], [57-217], [129-286], [220-259],
[68-274], [147-261], [30-223], [168-229], [102-218], [105-246], [131-165], [62-185], [179-237], [107-
131], [106-222], [161-200], [26-183], [184-260], [130-230], [128-270], [213-265], [148-214], [137-
269], [217-256], [108-262], [161-209], [171-224], [70-235], [63-74], [104-286], [29-89], [44-212],
[71-109], [217-223], [135-269], [35-114], [28-91], [27-139], [73-111], [125-222], [64-194], [128-271],
[106-270], [213-287], [61-180], [217-270], [107-243], [108-237], [234-287], [147-188], [63-239], [56-
276], [179-276], [238-241], [149-242], [248-285], [147-245], [62-276], [215-236], [70-120], [54-282],
[255-271], [48-146], [132-150], [243-275], [230-271], [44-90], [102-216], [71-169], [64-232], [65-

263], [104-261], [40-158], [89-216], [112-268], [60-171], [260-268], [211-218], [27-213], [197-260], [143-166], [198-253], [217-238], [213-284], [217-250], [129-287], [28-64], [32-288], [107-138], [26-236], [197-287], [249-266], [43-142], [167-203], [166-260], [140-280], [107-177], [161-235], [26-171], [118-213], [206-213], [26-90], [70-144], [42-216], [72-166], [263-266], [41-243], [151-243], [35-216], [33-151], [86-239], [148-177], [185-215], [115-128], [96-166], [96-156], [162-271], [61-217], [216-234], [105-134], [28-65], [68-266], [261-276], [174-252], [105-168], [27-136], [93-144], [33-269], [31-167], [126-266], [177-281], [204-219], [107-173], [126-164], [58-70], [127-260], [124-212], [163-223], [61-181], [31-214], [114-223], [153-238], [96-104], [70-238], [30-266], [125-240], [28-119], [121-251], [72-239], [169-218], [178-217], [51-232], [84-213], [116-169], [159-244], [152-215], [124-136], [165-269], [214-235], [122-135], [67-94], [68-193], [72-220], [76-116], [27-162], [232-236], [63-262], [104-245], [76-105], [178-185], [46-144], [72-141], [78-148], [167-272], [139-213], [37-220], [206-271], [79-237], [197-278], [92-271], [149-220], [129-166], [57-67], [26-220]

1.4 The preselected features of reactant/apo model

The numbers of preselected features of reactant/apo (1923) model is listed below as the format [%d1-%d2], %d1 and %d2 are the residue numbers of TEM1. They are the pairwise residues on TEM-1 used for reactant/apo training model in Random Forest.

[67-104], [105-157], [105-182], [85-105], [105-186], [105-193], [104-165], [104-184], [105-149], [98-215], [70-235], [105-242], [215-223], [182-287], [167-236], [40-287], [59-287], [33-68], [112-215], [238-287], [68-179], [70-253], [57-212], [130-283], [130-226], [62-105], [70-255], [68-143], [70-194], [100-229], [127-180], [171-249], [104-164], [104-225], [110-217], [104-237], [129-221], [68-161], [168-274], [69-179], [167-180], [148-275], [215-247], [36-68], [148-286], [169-237], [213-283], [167-223], [70-248], [212-246], [127-220], [131-216], [81-104], [98-224], [68-220], [70-286], [164-236], [66-170], [64-169], [105-259], [49-105], [63-105], [26-257], [102-222], [100-216], [32-268], [140-166], [70-184], [26-101], [67-105], [191-219], [69-253], [74-239], [71-242], [167-189], [70-234], [217-286], [66-104], [52-218], [68-105], [26-211], [76-105], [35-197], [143-215], [51-217], [67-70], [68-236], [165-179], [70-233], [109-287], [83-213], [166-262], [193-217], [37-163], [118-239], [236-260], [121-224], [69-165], [68-243], [166-252], [145-275], [214-224], [144-233], [136-215], [105-162], [129-217], [188-197], [71-124], [94-233], [120-274], [70-178], [104-167], [216-284], [236-277], [56-197], [89-186], [71-209], [105-234], [71-196], [75-105], [97-239], [141-163], [68-241], [70-257], [164-216], [150-271], [43-273], [212-238], [243-276], [75-147], [212-247], [72-239], [146-275], [78-192], [197-241], [164-217], [26-273], [151-276], [60-197], [78-105], [62-130], [170-196], [32-165], [102-218], [30-77], [37-166], [69-231], [165-259], [70-230], [68-217], [165-219], [106-241], [60-287], [172-225], [194-196], [26-214], [217-248], [170-245], [33-105], [103-224], [197-257], [163-270], [168-222], [239-287], [136-170], [217-224], [69-178], [243-274], [170-279], [170-183], [236-257], [218-222], [215-287], [131-176], [70-202], [216-234], [164-214], [106-165], [202-217], [68-163], [147-274], [237-259], [236-246], [70-101], [135-244], [60-166], [236-272], [107-222], [26-244], [105-197], [27-283], [215-250], [77-172], [234-237], [64-105], [80-219], [214-240], [218-250], [197-263], [72-109], [105-164], [35-271], [144-268], [70-204], [143-170], [213-218], [70-170], [210-237], [130-225], [105-222], [244-252], [26-202], [102-216], [83-240], [166-223], [165-263], [106-218], [132-164], [27-222], [145-217], [28-112], [215-280], [50-70], [79-152], [160-253], [261-274], [265-287], [41-228], [105-260], [169-271], [218-233], [234-239], [26-258], [77-274], [130-215], [169-189], [176-213], [70-185], [166-222], [70-183], [31-106], [67-271], [132-196], [218-221], [57-129], [128-178], [165-264], [89-184], [26-184], [217-242], [131-226], [61-171], [149-215], [86-186], [129-181], [105-188], [168-219], [26-259], [173-288], [70-251], [68-162], [106-236], [76-159], [27-236], [149-180], [212-237], [70-245], [167-231], [150-237], [27-60], [70-140], [120-239], [72-105], [209-238], [65-70],

[102-230], [135-196], [185-197], [73-130], [43-219], [153-216], [85-213], [57-282], [175-229], [186-238], [26-86], [27-279], [193-206], [179-216], [178-241], [217-246], [36-287], [103-236], [165-284], [71-235], [66-274], [217-227], [26-173], [173-255], [155-287], [125-240], [68-235], [30-276], [139-187], [144-272], [152-196], [64-155], [179-249], [30-220], [163-177], [88-201], [175-196], [69-104], [26-185], [162-267], [168-246], [78-130], [169-270], [170-255], [219-231], [67-134], [219-242], [27-278], [26-196], [29-214], [125-218], [216-219], [146-216], [31-236], [106-157], [118-198], [206-217], [162-284], [65-170], [213-220], [49-215], [105-244], [197-262], [105-137], [87-232], [172-237], [164-274], [196-266], [130-172], [164-170], [98-271], [27-161], [78-190], [163-216], [46-130], [104-149], [219-271], [64-106], [82-237], [196-277], [132-163], [68-106], [80-271], [165-232], [26-104], [146-270], [244-279], [174-216], [49-69], [142-189], [26-48], [69-287], [167-193], [52-197], [66-77], [69-258], [137-270], [122-237], [123-237], [245-275], [149-271], [219-275], [132-216], [145-267], [61-105], [216-248], [46-287], [84-174], [104-196], [162-216], [213-259], [70-231], [105-153], [27-215], [64-161], [147-216], [137-236], [148-268], [26-231], [262-277], [163-173], [199-238], [26-57], [236-276], [68-174], [218-223], [69-200], [215-248], [43-236], [70-267], [84-216], [54-105], [105-161], [168-220], [213-280], [26-224], [105-181], [44-105], [28-103], [121-192], [26-200], [122-249], [149-183], [218-279], [213-230], [65-129], [37-216], [134-176], [131-215], [33-107], [106-160], [101-237], [235-275], [129-238], [69-108], [69-216], [161-234], [83-270], [27-63], [163-217], [181-206], [26-117], [158-192], [219-274], [27-178], [105-262], [28-122], [27-268], [56-130], [215-222], [71-213], [31-172], [243-271], [58-105], [160-243], [26-235], [197-259], [177-287], [113-243], [142-186], [91-182], [165-196], [48-165], [140-248], [71-83], [163-274], [26-283], [105-208], [73-98], [87-187], [139-277], [43-149], [40-112], [194-239], [173-222], [26-272], [69-269], [173-258], [236-262], [106-163], [173-229], [106-181], [51-166], [27-264], [122-178], [236-256], [236-287], [133-162], [35-266], [107-242], [90-272], [87-199], [77-219], [245-269], [71-187], [69-240], [72-280], [91-237], [104-161], [177-272], [37-213], [217-234], [137-239], [186-218], [90-169], [68-256], [70-240], [149-239], [103-282], [130-178], [245-285], [132-161], [69-243], [27-102], [167-188], [168-207], [37-142], [227-241], [145-274], [57-104], [74-145], [103-272], [76-130], [26-269], [176-244], [76-269], [64-160], [124-166], [101-219], [29-167], [62-217], [74-217], [233-238], [120-277], [51-287], [213-278], [168-243], [28-110], [36-106], [35-287], [106-263], [216-233], [216-222], [138-168], [226-242], [120-178], [26-210], [175-227], [263-271], [169-257], [168-236], [77-130], [165-234], [35-288], [125-238], [131-236], [160-235], [42-287], [61-197], [68-245], [105-215], [181-213], [67-170], [27-157], [104-200], [139-215], [38-257], [69-217], [159-217], [29-56], [26-43], [111-282], [209-281], [150-217], [37-54], [136-219], [44-236], [81-134], [197-245], [34-164], [36-237], [163-268], [68-223], [26-276], [149-216], [26-282], [201-284], [103-220], [145-245], [163-235], [161-266], [165-235], [37-280], [216-279], [49-218], [85-238], [218-252], [143-271], [86-197], [38-85], [105-241], [75-237], [66-76], [103-213], [87-185], [34-104], [69-239], [101-215], [111-274], [81-216], [142-185], [167-217], [113-237], [64-150], [68-265], [68-148], [193-216], [106-171], [79-184], [74-165], [80-159], [71-270], [50-72], [118-219], [147-278], [143-176], [105-209], [117-184], [85-274], [98-218], [85-248], [122-259], [243-279], [104-198], [183-196], [220-286], [31-88], [69-172], [36-126], [46-236], [26-51], [119-184], [181-196], [64-145], [107-178], [104-244], [70-274], [132-169], [70-242], [178-193], [229-273], [26-70], [69-257], [143-276], [90-190], [28-275], [59-176], [105-243], [43-81], [163-178], [30-169], [140-239], [180-210], [30-164], [79-172], [59-213], [109-245], [26-192], [220-260], [80-239], [84-184], [264-287], [73-191], [197-248], [26-254], [46-253], [29-213], [217-250], [31-60], [151-282], [31-165], [180-271], [234-238], [126-284], [26-285], [107-196], [72-181], [42-132], [131-237], [213-236], [133-264], [26-253], [62-153], [67-119], [39-221], [105-112], [156-171], [249-252], [33-64], [82-129], [115-217], [67-69], [66-281], [39-75], [26-124], [42-177], [58-276], [84-236], [170-248], [121-173], [136-234], [143-272], [71-239], [87-268], [212-214], [70-74], [129-158], [236-261], [127-184], [69-221], [134-236], [105-141], [108-188], [219-221], [217-229], [64-147], [70-243], [100-234], [165-277], [98-209], [67-247], [71-190], [131-180], [211-219], [216-224], [30-38], [109-215], [28-274],

[27-51], [135-222], [102-235], [40-182], [236-268], [109-274], [126-239], [27-66], [157-216], [26-106], [205-277], [79-271], [64-104], [48-69], [91-166], [134-159], [26-288], [145-196], [33-267], [26-256], [91-174], [219-269], [61-216], [26-156], [26-267], [178-233], [190-217], [126-265], [204-218], [169-264], [48-95], [150-185], [98-219], [105-236], [30-75], [49-93], [175-228], [35-213], [60-173], [146-196], [147-277], [139-269], [136-216], [190-214], [105-167], [71-201], [158-178], [160-216], [70-241], [92-138], [71-112], [171-186], [234-267], [117-206], [37-47], [63-213], [47-71], [175-269], [162-268], [105-183], [69-260], [70-252], [26-159], [36-260], [67-90], [78-156], [236-249], [106-235], [55-85], [105-160], [148-274], [60-276], [215-219], [236-288], [109-275], [239-279], [31-265], [83-92], [95-213], [88-137], [212-252], [104-247], [172-262], [205-216], [26-212], [141-234], [160-193], [175-225], [206-270], [144-270], [70-163], [182-263], [70-239], [70-210], [137-197], [26-171], [172-197], [70-268], [113-157], [104-133], [105-178], [154-271], [49-70], [28-223], [37-172], [107-200], [131-182], [34-147], [86-237], [139-271], [150-245], [32-240], [216-249], [132-223], [166-216], [107-170], [160-174], [107-215], [93-236], [116-172], [105-132], [26-118], [130-162], [47-216], [165-216], [58-217], [71-129], [37-211], [174-192], [263-269], [142-180], [92-136], [177-186], [47-81], [65-287], [52-233], [61-83], [62-128], [97-176], [69-174], [170-261], [86-124], [129-180], [69-184], [61-75], [44-114], [80-216], [37-71], [165-174], [143-185], [243-283], [68-118], [190-213], [63-106], [65-104], [123-271], [104-236], [104-109], [56-196], [128-161], [113-150], [153-233], [69-153], [244-276], [122-261], [239-259], [162-245], [132-262], [59-236], [52-217], [108-260], [51-212], [69-259], [166-212], [248-254], [131-218], [80-240], [218-227], [83-168], [173-274], [218-286], [51-120], [201-219], [42-183], [237-275], [70-214], [187-279], [38-51], [69-72], [144-169], [29-104], [75-219], [238-247], [173-227], [125-260], [101-270], [164-250], [166-219], [49-73], [166-224], [39-82], [52-166], [26-207], [182-272], [35-117], [70-222], [35-82], [208-217], [29-131], [214-270], [80-254], [157-222], [149-267], [84-274], [264-288], [136-222], [213-241], [172-212], [244-274], [68-152], [165-180], [215-231], [68-166], [126-225], [86-156], [161-189], [152-220], [264-282], [243-282], [30-59], [48-216], [27-275], [212-216], [219-233], [222-233], [159-239], [159-279], [80-217], [173-191], [63-286], [146-271], [184-215], [26-245], [148-183], [236-251], [40-197], [73-150], [92-203], [26-188], [147-204], [217-241], [52-212], [118-196], [27-160], [236-274], [29-223], [57-217], [114-283], [153-182], [167-191], [145-268], [40-285], [87-125], [238-260], [91-216], [26-201], [95-158], [166-261], [194-286], [215-236], [217-237], [172-226], [105-154], [177-273], [216-287], [187-233], [87-139], [36-149], [78-234], [51-76], [70-226], [163-206], [85-197], [85-113], [27-38], [245-271], [79-218], [136-154], [63-246], [69-282], [70-137], [67-250], [26-134], [71-77], [191-197], [169-252], [103-241], [69-186], [195-197], [45-82], [32-110], [47-177], [106-215], [217-225], [73-258], [126-216], [119-223], [32-213], [91-222], [54-196], [159-187], [179-208], [71-195], [28-111], [213-219], [37-288], [42-256], [73-115], [56-117], [73-236], [242-278], [171-218], [27-251], [215-245], [138-243], [143-166], [47-236], [219-237], [98-176], [27-182], [58-70], [79-219], [244-282], [28-131], [125-280], [110-270], [153-278], [251-288], [75-111], [103-207], [100-191], [142-287], [208-242], [182-245], [117-210], [28-115], [74-131], [41-145], [76-235], [69-263], [212-280], [105-136], [81-196], [112-164], [93-226], [245-276], [67-274], [103-218], [178-222], [104-220], [97-182], [195-230], [62-144], [26-193], [183-221], [95-216], [134-163], [26-177], [27-62], [166-246], [188-214], [73-284], [187-188], [219-272], [186-224], [39-218], [58-274], [145-239], [34-105], [67-235], [99-106], [90-197], [76-196], [26-168], [153-272], [219-250], [106-238], [181-267], [135-236], [58-82], [183-271], [181-199], [52-75], [90-214], [204-215], [29-38], [220-243], [60-213], [87-216], [106-271], [26-72], [50-178], [59-254], [84-275], [90-266], [57-175], [31-44], [163-176], [40-72], [149-240], [126-288], [43-66], [238-276], [102-238], [70-177], [93-177], [176-232], [104-158], [212-217], [240-268], [48-64], [94-104], [124-217], [140-251], [29-275], [78-128], [47-235], [167-260], [67-220], [52-219], [74-166], [117-197], [254-264], [171-258], [44-173], [40-151], [68-153], [105-258], [110-167], [44-149], [88-109], [212-282], [218-226], [152-210], [102-118], [243-277], [246-275], [216-261], [234-279], [70-278], [167-253],

[64-262], [215-233], [131-224], [57-225], [161-236], [40-226], [99-220], [34-49], [47-166], [73-248], [168-173], [91-283], [34-70], [67-244], [199-243], [159-216], [125-182], [107-282], [27-217], [178-266], [196-247], [110-244], [169-280], [73-156], [70-105], [70-93], [58-197], [235-268], [176-284], [80-180], [38-288], [39-166], [256-276], [58-259], [65-108], [94-257], [66-213], [70-110], [244-286], [77-216], [193-215], [217-247], [34-51], [252-264], [168-215], [68-129], [193-260], [90-225], [29-217], [171-237], [80-188], [59-197], [208-248], [44-166], [104-116], [166-263], [26-163], [91-204], [43-226], [217-280], [175-253], [67-130], [155-214], [230-271], [66-288], [173-248], [38-231], [26-194], [30-187], [70-216], [34-214], [39-270], [116-226], [27-116], [156-182], [192-235], [35-113], [161-279], [104-238], [160-186], [71-104], [202-216], [28-219], [97-273], [26-36], [239-274], [227-239], [265-284], [34-161], [54-117], [133-188], [142-228], [120-235], [86-123], [162-275], [121-208], [105-134], [165-245], [71-117], [84-213], [80-195], [29-234], [41-190], [89-176], [152-183], [73-244], [131-247], [263-274], [118-174], [186-275], [26-180], [216-252], [164-202], [193-283], [91-132], [139-242], [109-216], [33-62], [162-175], [104-160], [110-194], [36-218], [103-257], [129-223], [222-282], [284-287], [99-105], [83-273], [105-185], [44-104], [66-173], [198-249], [115-178], [130-181], [60-272], [46-136], [144-288], [59-194], [119-227], [192-238], [130-163], [42-219], [179-268], [77-159], [103-118], [218-224], [80-84], [238-288], [107-176], [42-216], [198-257], [71-200], [107-154], [186-239], [41-217], [58-98], [174-197], [236-279], [63-128], [150-277], [237-264], [244-270], [106-282], [132-189], [106-212], [105-220], [66-90], [105-171], [31-167], [40-262], [91-116], [164-175], [103-209], [57-159], [54-213], [40-274], [160-270], [151-240], [114-256], [174-219], [52-144], [70-200], [122-133], [154-174], [174-212], [149-268], [47-256], [63-182], [193-262], [82-157], [34-198], [96-189], [64-168], [167-251], [143-168], [29-103], [28-139], [142-184], [143-216], [127-268], [52-256], [128-136], [69-234], [69-161], [31-118], [200-207], [83-111], [208-284], [70-272], [264-276], [218-247], [249-280], [65-178], [145-153], [40-283], [90-260], [194-275], [45-196], [111-220], [95-204], [198-201], [165-220], [133-282], [59-147], [172-261], [141-236], [121-156], [39-61], [66-82], [35-215], [177-183], [26-287], [68-151], [126-180], [169-173], [112-113], [179-276], [161-166], [50-215], [151-185], [176-183], [33-141], [176-264], [27-40], [271-280], [56-280], [84-214], [64-287], [55-70], [86-125], [103-249], [48-145], [236-259], [184-237], [74-103], [26-30], [39-188], [35-216], [67-260], [252-269], [89-222], [38-131], [31-129], [33-287], [145-216], [33-213], [157-210], [26-268], [177-266], [177-215], [137-156], [147-217], [170-197], [59-137], [129-192], [70-82], [144-218], [160-209], [26-232], [220-284], [122-236], [118-283], [142-270], [139-270], [88-152], [26-154], [250-267], [180-217], [181-279], [116-215], [133-219], [144-275], [32-89], [176-192], [108-216], [95-225], [230-252], [71-127], [128-162], [156-180], [37-197], [85-226], [73-218], [72-130], [199-263], [94-174], [67-216], [165-243], [106-243], [28-133], [161-228], [216-236], [37-214], [160-288], [117-274], [103-278], [42-89], [36-169], [47-104], [138-285], [133-261], [44-283], [77-205], [115-240], [42-243], [160-269], [68-216], [129-160], [164-194], [167-284], [141-157], [82-132], [174-265], [125-136], [120-208], [28-224], [92-183], [54-120], [112-206], [68-125], [242-287], [200-236], [108-117], [102-195], [117-119], [175-237], [76-270], [166-275], [188-285], [65-233], [26-33], [36-173], [136-220], [170-182], [94-160], [62-96], [69-254], [29-251], [84-178], [114-282], [92-213], [70-136], [68-108], [118-161], [68-128], [104-242], [68-272], [100-221], [45-96], [241-275], [46-178], [61-228], [59-91], [26-42], [80-105], [39-257], [117-171], [150-248], [171-195], [200-286], [55-108], [86-127], [35-77], [33-89], [187-216], [31-288], [164-244], [167-192], [116-239], [77-265], [216-251], [261-284], [154-190], [113-223], [107-157], [81-103], [158-188], [107-130], [56-143], [198-224], [89-149], [47-106], [55-138], [50-90], [117-240], [68-214], [59-165], [255-274], [85-278], [65-250], [104-203], [225-273], [145-213], [105-172], [57-283], [236-285], [60-193], [67-151], [40-266], [52-127], [35-185], [177-195], [104-136], [196-252], [218-229], [72-275], [62-182], [48-212], [74-83], [34-138], [71-268], [32-45], [80-212], [49-283], [77-158], [203-236], [82-159], [168-231], [149-214], [165-275], [180-193], [165-237], [188-215], [104-227], [91-240], [28-228], [94-146], [61-98], [87-132], [151-277], [112-202], [38-137], [39-154], [239-272], [101-235], [153-236], [75-130], [131-174], [66-270], [195-232],

[67-136], [101-202], [122-181], [63-176], [79-244], [81-252], [200-235], [64-288], [34-266], [70-232], [87-274], [170-212], [79-130], [172-270], [36-288], [88-147], [213-248], [43-234], [166-244], [26-32], [35-72], [27-47], [87-143], [163-229], [82-270], [74-173], [162-225], [172-230], [45-274], [92-152], [35-95], [73-147], [141-196], [44-129], [123-259], [83-253], [128-238], [58-280], [141-224], [125-181], [36-240], [116-234], [56-277], [93-213], [105-248], [39-282], [30-192], [26-221], [116-122], [55-125], [65-185], [216-275], [79-149], [65-163], [116-240], [251-266], [167-280], [134-202], [220-238], [97-102], [116-221], [27-39], [93-155], [108-285], [87-179], [243-273], [70-125], [78-85], [33-219], [47-145], [49-71], [26-249], [27-85], [166-248], [216-281], [215-224], [105-150], [163-192], [70-282], [99-284], [53-70], [106-225], [133-152], [154-175], [105-173], [171-263], [67-166], [57-267], [124-260], [71-223], [41-242], [115-275], [26-103], [88-154], [70-78], [26-213], [112-144], [196-230], [165-261], [57-194], [53-117], [122-220], [96-103], [113-216], [163-214], [47-252], [50-136], [87-155], [64-163], [58-84], [47-232], [100-213], [160-231], [74-206], [129-132], [74-81], [233-279], [173-286], [46-248], [126-268], [100-190], [129-171], [66-196], [89-197], [234-275], [118-222], [106-159], [67-165], [128-260], [64-177], [70-167], [199-281], [32-123], [251-259], [233-287], [31-234], [88-270], [81-198], [84-209], [129-156], [152-240], [29-69], [129-170], [49-60], [107-144], [105-286], [104-262], [66-160], [193-235], [146-166], [43-265], [28-132], [64-185], [196-231], [266-269], [129-255], [36-272], [40-261], [255-269], [31-100], [125-215], [147-280], [163-272], [225-258], [216-220], [29-170], [217-221], [97-198], [59-279], [62-213], [104-243], [179-271], [93-151], [61-207], [40-288], [214-250], [70-224], [125-276], [160-217], [175-181], [63-113], [72-207], [128-219], [38-166], [139-216], [88-234], [193-223], [63-153], [119-276], [143-152], [45-236], [72-143], [129-246], [90-205], [81-192], [150-199], [69-238], [88-236], [101-231], [183-224], [149-192], [125-201], [67-107], [136-178], [217-265], [41-107], [110-237], [147-163], [106-214], [99-267], [47-74], [81-158], [186-271], [147-223], [46-183], [129-229], [78-274], [71-286], [72-237], [36-127], [105-127], [45-237], [164-243], [92-177], [120-141], [159-275], [44-220], [243-270], [137-189], [47-175], [217-230], [76-189], [69-130], [214-223], [172-219], [111-272], [111-153], [123-161], [71-207], [86-171], [157-206], [153-270], [155-190], [166-286], [95-240], [145-221], [55-87], [142-143], [136-212], [74-147], [159-221], [37-118], [196-287], [177-236], [69-274], [104-249], [69-164], [40-224], [162-266], [188-242], [78-241], [135-156], [245-279], [87-197], [111-214], [61-194], [145-233], [127-164], [69-102], [159-248], [101-278], [108-273], [52-284], [202-214], [77-223], [81-105], [125-242], [92-235], [203-239]

2 Supplementary Figures and Tables

2.1 Supplementary Figures

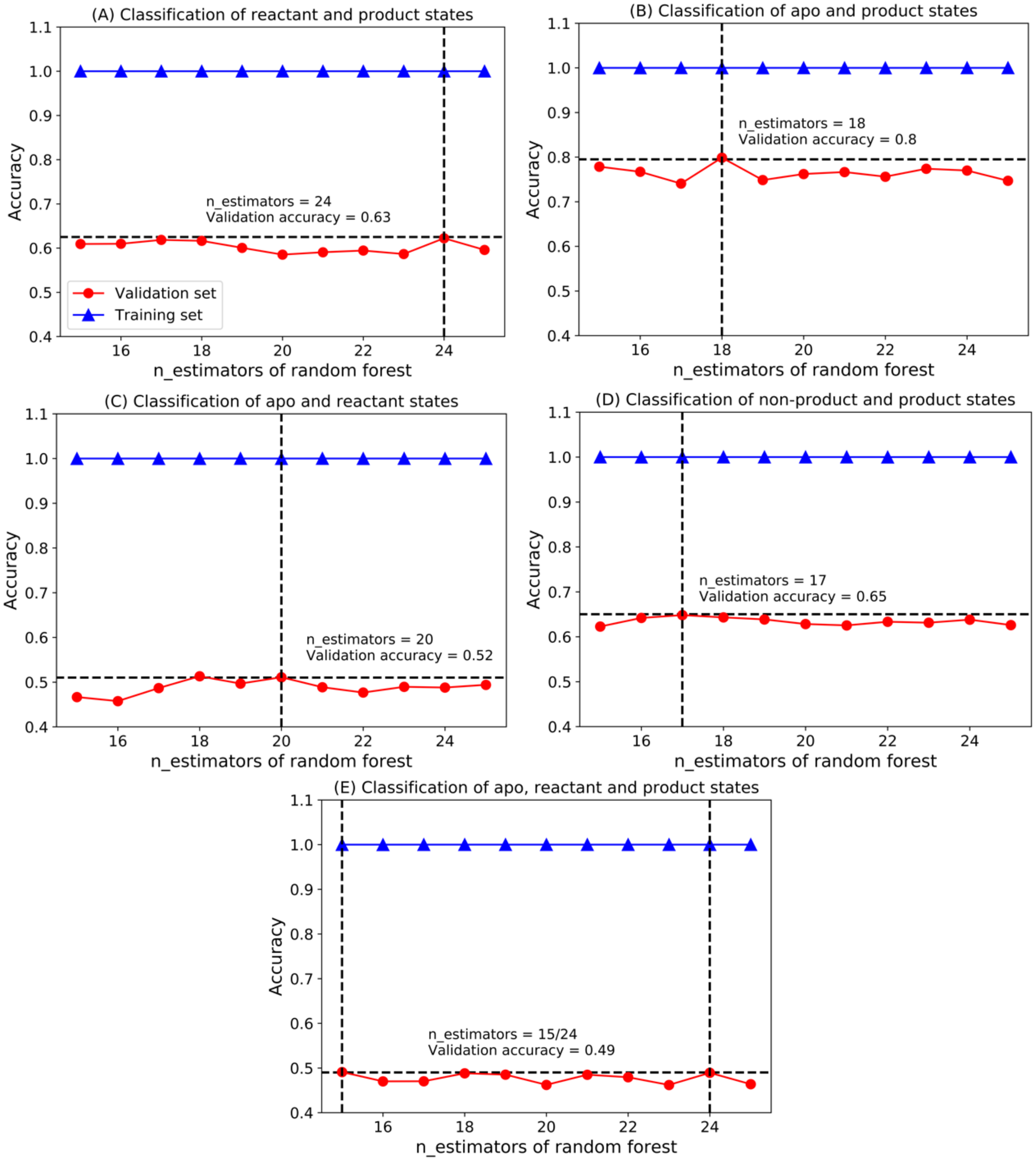

Supplementary Figure 1. The accuracy of validation set and training set of TEM-1 in the classifications: (A) reactant and product states; (B) apo and product states; (C) apo and reactant states; (D) non-product and product states; (E) apo, reactant and product states using the random forest method. The x-axis represents the number of estimators (the number of trees used in random forest) used in random forest method; the y-axis represents the accuracy of each model. The number of estimators with the highest validation accuracy is the parameter used in the corresponding classification model.

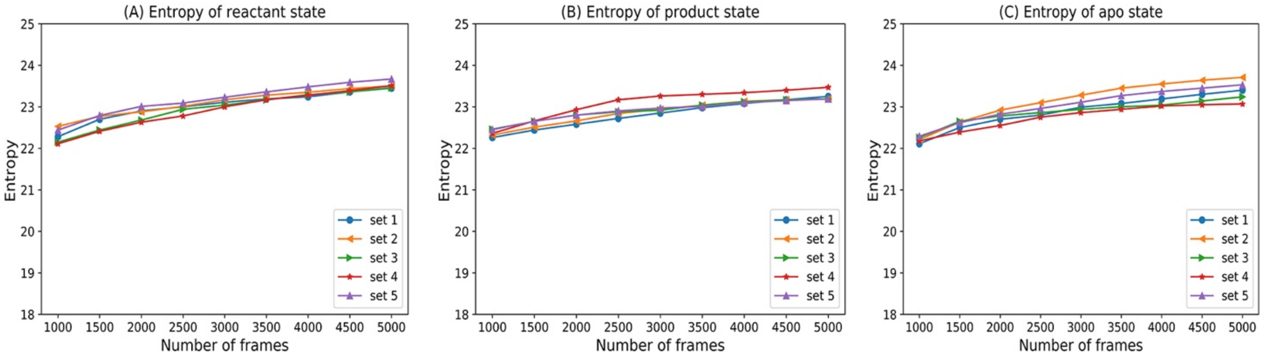

Supplementary Figure 2. The entropy of each state in five independent simulations (set1 – set5): (A) reactant state, (B) product state, (C) apo state. All three states display convergence of simulations towards the 100 ns simulations.

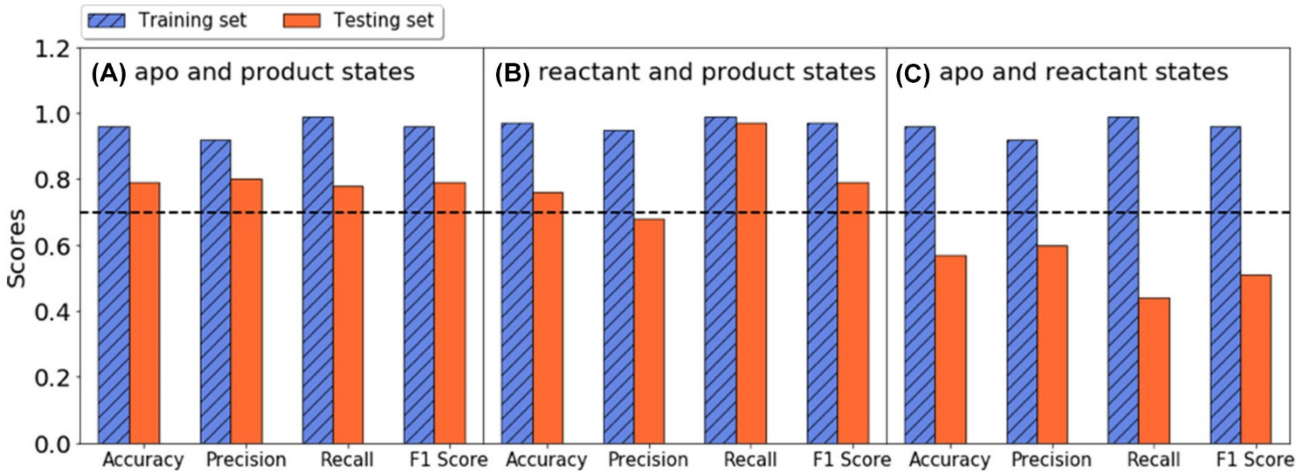

Supplementary Figure 3. The accuracy, precision, recall and F1 scores of training set and testing set of TEM-1 in the classifications: (A) apo and product states; (B) reactant and product states; (C) apo and reactant states using the neural networks method. The four scores are calculated by same methods stated in the main text.

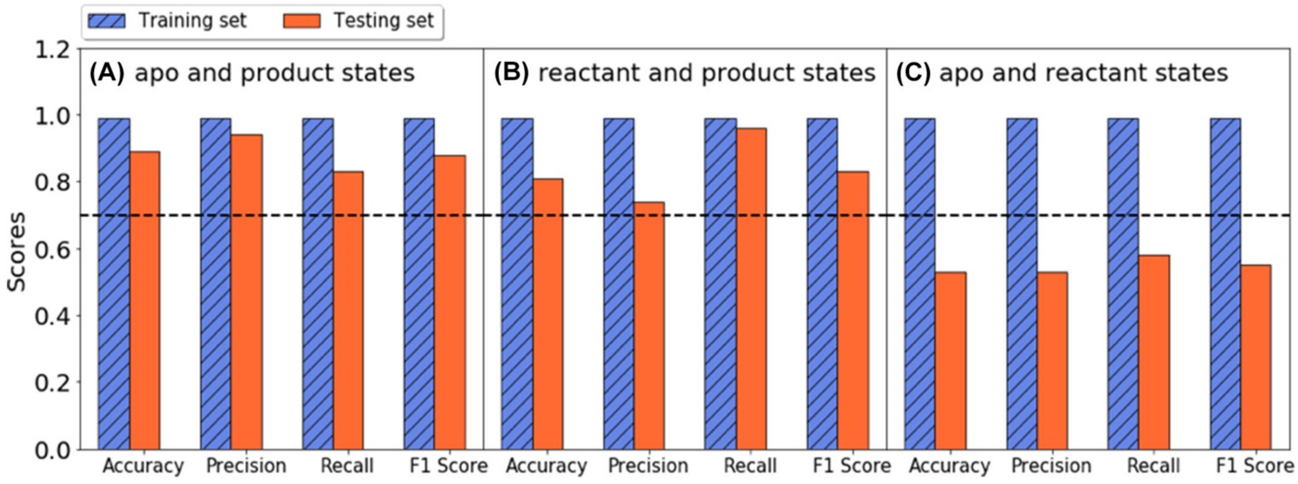

Supplementary Figure 4. The accuracy, precision, recall and F1 scores of training set and testing set of TEM-1 in the classifications model using the support vector machine method: (A) apo and product states; (B) reactant and product states; (C) apo and reactant states. The four scores are calculated by same methods stated in the main text.

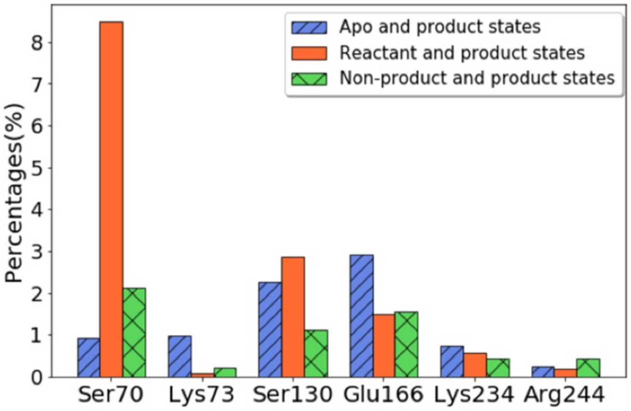

Supplementary Figure 5. Key residues (Ser70, Lys73, Ser130, Glu166, Lys234 and Arg244) at TEM-1 active site with significant feature importance in three classification models: apo/product model (blue bars), reactant/product model (red bars) and non-product/product model (green bars).

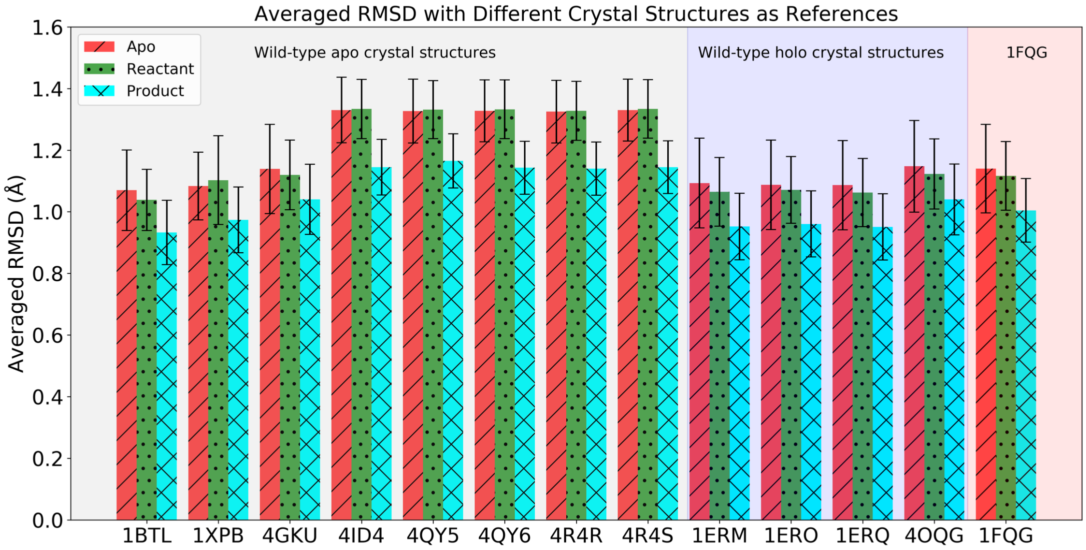

Supplementary Figure 6. The averaged RMSDs with different crystal structures as references for apo (red color background with single slash), reactant (green color background with dots) and product (cyan color background with crossed slash) states simulations. There are three groups of crystal structures: wild-type apo crystal structures in gray background part with PDB IDs labeled in X-axis (1BTL, 1XPB, 4GKU, 4ID4, 4QY5, 4QY6, 4R4R, 4R4S), wild-type holo crystal structures in light blue background part with PDB IDs labeled in X-axis (1ERM, 1ERO, 1ERQ, 4OQG) and the 1FQG crystal structure in pink background part. All the RMSD results listed were calculated using VMD 1.9.4. As an initial step for calculating the RMSD, a crystal structure is aligned to the trajectories of apo, reactant and product states simulations using the stamp structure alignment tool. The RMSD results are generated by RMSD visualizer tool with each crystal structure as a reference. Both the tools for alignment and RMSD visualizer are built in VMD 1.9.4. The RMSD for total 25000 frames of trajectories from 5 independent simulations of each state are calculated. The error bars show the standard deviation for the averaged RMSD values.
